# Integrative transcriptomics and reverse network pharmacology prioritize DPP4 and *Achyranthes bidentata* in Barrett’s esophagus

**DOI:** 10.1371/journal.pone.0359324

**Published:** 2026-09-25

**Authors:** Jiaying Wu, Zhen Zheng, Jiaming Wu

**Affiliations:** 1 School of Nursing, Ningbo College of Health Sciences, Ningbo, Zhejiang Province, China; 2 Department of Chemoradiation Oncology, The Affiliated Lihuili Hospital of Ningbo University, Ningbo, Zhejiang Province, China; University of Central Florida College of Medicine, UNITED STATES OF AMERICA

## Abstract

Barrett's esophagus (BE) is a precursor of esophageal adenocarcinoma, but candidate expression markers and preventive hypotheses require evaluation across patients, cohorts, and evidence levels. We analyzed two GPL96 discovery cohorts comprising 15 matched normal–BE pairs, used pair-aware differential modeling and resampling, and rebuilt four external BE-tissue assessments from official GEO files. The primary analysis yielded 1,085 differentially expressed genes; LASSO selected 13 features, linear SVM-RFE selected 6, and pair-bootstrap Random Forest retained 20. DPP4 and CCRL2 were shared by the three primary feature sets, but not after phenotype-preserving ComBat sensitivity analysis. External Affymetrix AUCs were 1.000, 0.855, and 0.976 for DPP4 and 1.000, 0.858, and 0.963 for CCRL2. In the Illumina GSE28302 cohort, fixed-direction DPP4 AUC was 0.444 and CCRL2 lacked a uniquely mapped probe. Seventy-nine DEGs intersected the verified 710-gene disease union; STRING/CTD analysis ranked Niuxi, Mahuang, and Mahuanggen first through third. Reference-controlled docking advanced kaempferol–PPARG as the primary structural hypothesis and taxifolin–NQO1 as a secondary extension. Twelve 100 ns trajectories retained pocket-region contacts, but query compounds showed greater pose rearrangement than the reference ligands. These findings prioritize candidates for experimental validation and do not establish a diagnostic test, direct binding, expression reversal, or therapeutic efficacy.

## 1. Introduction

Barrett's esophagus (BE) is a premalignant condition characterized by the replacement of the normal stratified squamous epithelium of the distal esophagus with a metaplastic columnar epithelium [1]. This metaplastic transition is the primary precursor lesion for esophageal adenocarcinoma (EAC), a highly aggressive malignancy with a rapidly increasing incidence and a five-year survival rate of less than 20% [2]. The progression from BE to EAC follows a well-defined metaplasia-dysplasia-carcinoma sequence, primarily driven by chronic gastroesophageal reflux disease and subsequent mucosal injury [3,4]. Current clinical management relies heavily on endoscopic surveillance and acid suppression therapy; however, these approaches are limited by sampling error, high costs, and an inability to reliably predict or halt neoplastic progression in individual patients [3,4]. Consequently, identifying the early molecular drivers of BE and developing targeted interventions to disrupt this sequence remain critical clinical imperatives.

The pathogenesis of BE involves transcriptomic remodeling across epithelial and microenvironmental compartments. Chronic reflux-associated acid and bile exposure can promote inflammatory and wound-repair programs during metaplastic remodeling [5–7]. Machine-learning feature-screening methods can prioritize expression features, but paired sampling, preprocessing specifications, and grouped resampling must be respected to avoid patient-level leakage and unstable intersections [8–10].

Identifying candidate biomarkers and generating pharmacological hypotheses are separate objectives. In the gene-to-herb workflow used here, disease-associated genes were intersected with DEGs, central nodes were ranked in a protein-interaction network, CTD chemicals associated with those nodes were retrieved, and herbs enriched for the retrieved chemicals were identified. The term reverse network pharmacology describes this disease-gene-to-chemical-to-herb search direction; it does not mean that a chemical reverses the direction of a DEG. The workflow is sensitive to database coverage and cannot establish compound exposure, botanical exclusivity, efficacy, or safety [11–16]. For botanical interpretation, Niuxi (*Achyranthes bidentata* root) provides a traditional-use context of promoting blood circulation and relieving stasis; any connection to reflux-associated mucosal injury or remodeling requires a separate pharmacological assessment [17].

This study addressed three exploratory aims: (i) to perform pair-aware screening and multi-cohort assessment of BE-associated expression features using three complementary machine-learning methods and a preprocessing-sensitivity analysis; (ii) to prioritize gene-to-chemical-to-herb associations through a source-audited reverse network-pharmacology workflow; and (iii) to evaluate the dynamic stability of structurally eligible compound–target hypotheses through reference-controlled molecular docking followed by three independently seeded 100 ns MD replicas per retained complex.

## 2. Materials and Methods

### 2.1 Data acquisition and preprocessing

GEO microarray expression data were analyzed for Barrett’s esophagus (BE) and normal esophageal squamous tissue. The discovery cohort comprised seven matched normal–BE pairs from GSE13083 (GPL96; non-dysplastic BE) and eight pairs from GSE1420 (GPL96). Small-intestinal samples in GSE13083 and adenocarcinoma samples in GSE1420 were excluded. GSE1420 was derived from patients with Barrett-associated adenocarcinoma, and dysplasia status of its BE samples was not uniformly annotated. External assessments used GSE36223 (GPL571; 23 pairs: 17 without dysplasia, two low-grade and four indefinite), GSE39491 (GPL571; 40 pairs, dysplasia incompletely annotated), GSE26886 (GPL570; 19 healthy normal and 20 BE samples, unpaired, dysplasia unstated), and GSE28302 (GPL2507; nine normal and 22 non-dysplastic BE samples, unpaired). GSM inclusion lists and available clinical metadata are provided in Table A in S1 Data.

Discovery matrices were matched by gene symbol after official GPL96 annotation, duplicate-symbol averaging and correction of spreadsheet-converted symbols. The primary preprocessing specification used parametric ComBat with GEO study as batch and no phenotype covariate; a sensitivity analysis included phenotype in the ComBat design [18]. External cohorts were processed separately using depositor-supplied values: GCRMA/log2 for GSE36223, RMA for GSE39491, GC-RMA for GSE26886, and log2 background-corrected Lowess normalization for GSE28302. Probes without a unique gene symbol were excluded, and multiple probes for a symbol were averaged with limma::avereps. External cohorts were not merged or subjected to additional cross-cohort normalization. GSE77563 was ineligible for BE-versus-normal assessment because it contained only histologically normal squamous tissue. Mapping details are supplied in Tables L and M in S1 Data.

Batch association was summarized by the partial R^2^ attributable to study after conditioning on phenotype: (RSSreduced − RSSfull)/RSSreduced. Global partial R^2^ used sums of residual sums of squares across genes; gene-level distributions and variance-weighted values for the first ten principal components were also calculated. For 999 permutations, study labels were reassigned between complete patient pairs while retaining the original numbers of pairs per study; both samples from a patient always received the same label. Empirical P values were (1 + number of permuted statistics at least as large as observed)/1,000. These comparisons characterize study association in the fixed matrices and do not establish preservation of biological signal (Table H in S1 Data; Methods in S1 File).

This study used public de-identified data and involved no recruitment or access to identifiable records. No new participant consent or institutional ethics approval was required for this secondary analysis; source-study information is available in the GEO records and publications.

### 2.2 Differential expression and functional enrichment

Differential expression was assessed with limma using subject-pair and tissue-condition terms, followed by empirical Bayes moderation [19]. Genes with |log2 fold change| > 1 and BH-adjusted P < 0.05 were retained. Gene symbols were mapped to Entrez identifiers using org.Hs.eg.,db, with official-symbol mapping followed by alias lookup; ambiguous mappings were excluded and Entrez identifiers deduplicated. GO and KEGG over-representation analyses used clusterProfiler and the same mapping procedure for foreground and background genes [20,21]. The background comprised all genes tested for differential expression that could be mapped unambiguously and were annotated in the respective database. Gene sets containing 10–500 background genes were tested. GO biological process, cellular component and molecular function were analyzed with BH correction within each ontology; KEGG used the archived human pathway snapshot dated 4 September 2026 and BH correction across tested pathways. Terms with nominal P < 0.05 and BH-adjusted P < 0.05 were retained. Study-specific gene-to-Entrez mappings, background sets and tested-term results are supplied in S1 Data; raw KEGG snapshots are excluded from the public archive.

### 2.3 Machine-learning feature selection and external assessment

Three feature-selection methods were applied to the discovery DEG matrix. LASSO logistic regression used standardized predictors, five-fold cross-validation grouped by patient, and non-zero coefficients at lambda.min [22]. Linear SVM-RFE ranked predictors by squared weights within each of five patient-grouped outer training folds; four grouped inner folds tuned cost over 2^(−6:6). The feature count minimizing mean outer-fold error over 1–30 features was selected, and final genes were ordered by mean outer-training-fold rank [23]. Random Forest used 100 bootstrap resamples of complete patient pairs and 1,000 trees per resample. The final 20 genes were ordered by the frequency of appearing in each forest’s top 20, with mean normalized Gini importance as a tie-breaker [24]. Genes shared by all three methods were carried forward. The workflow was repeated using phenotype-preserving ComBat. Parameter and seed details are provided in Table B in S1 Data and Methods in S1 File.

ComBat and DEG filtering were performed on the full discovery cohort before resampling. SVM rankings were held fixed during inner cost tuning, and outer-fold errors were used to select feature count. Accordingly, the resampling results describe feature selection conditional on this preprocessing, rather than unbiased end-to-end predictive validation.

Candidate expression was assessed within each external cohort using ROC AUC, with direction fixed to higher expression in BE. 95% confidence intervals used 5,000 complete-pair bootstrap resamples for paired cohorts and DeLong’s method for unpaired cohorts. AUCs were not pooled. No transportable threshold or calibrated diagnostic probability model was developed; therefore, calibration and decision-curve analyses were not undertaken. DPP4-associated pathway and immune analyses were designated exploratory functional follow-up.

### 2.4 DPP4-associated pathways and immune-cell fractions

GSEA compared BE samples below versus at or above the median DPP4 expression. Genes with positive mean discovery-cohort expression were ranked by the difference in mean log2 expression between groups. The archived MSigDB KEGG_LEGACY GMT file was analyzed with clusterProfiler GSEA/fgsea using gene-set sizes 10–500, exponent 1, eps = 10^−10^, nPermSimple = 10,000, seed 20260906, and serial execution; BH correction covered all tested terms [25]. Plotting used running-score curves with gene-hit tracks and the ranked-list metric; displayed terms and statistical summaries are archived.

Relative fractions of 22 immune-cell types were estimated with the local CIBERSORT v1.03 implementation and LM22 signature [26]. The ComBat expression matrix was converted internally from log2 to linear values and quantile-normalized (QN = TRUE). Deconvolution used perm = 0, so no permutation-based sample-quality P values were available; all discovery samples were retained, and correlations were evaluated within BE samples. The exact invocation, input hashes and numerical reproduction check are provided in Methods in S1 File and S1 Data.

Simple Spearman correlations related DPP4 to regulatory T-cell, resting mast-cell, activated-memory CD4 T-cell and M0 macrophage fractions. An epithelial score was the mean of standardized KRT8, EPCAM, KRT18 and KRT19 expression. Partial Spearman correlations were Pearson correlations between residuals obtained by regressing ranked DPP4 and ranked cell fractions on the ranked epithelial score; average ranks were used for ties, and approximate two-sided P values used n − 3 degrees of freedom. BH correction was applied separately to the four simple and four adjusted tests. This analysis was exploratory, and correction was limited to these four cell types. The sum of relative fractions was not used as a covariate because it does not measure absolute immune infiltration.

### 2.5 Protein-interaction network and hub ranking

BE-associated gene lists from GeneCards, OMIM and CTD were merged and intersected with the discovery DEGs. Source-file hashes, selected gene outputs and access information are supplied in S1 Data; restricted raw exports are not redistributed, and historical search dates and database-specific score thresholds could not be reconstructed. Intersecting genes were queried against the STRING 12.0 human functional-association network on 4 September 2026, using a minimum combined score of 0.4 and no added interactors [27,28]. Unique undirected edges were used to calculate node degree. The top 30 degree-ranked genes were carried forward for chemical retrieval; degree was the sole ranking criterion.

### 2.6 Reverse network pharmacology

Human chemical–gene records for the top 30 hubs were extracted from the archived CTD interaction file [29]. Unique chemical names were matched to the herb–ingredient table provided as the raw input in S1 Data (refer.Drug.csv). Herb enrichment used a one-sided hypergeometric test with all ingredients in that table as background, considering herb sets of 5–500 ingredients. BH correction covered all eligible herb sets, including those with no overlap; nominal P < 0.05 and adjusted P < 0.05 defined retained herbs. Herb–ingredient–target networks were assembled from the matched records. CTD action annotations were retained as context-dependent associations. This workflow does not assess oral absorption, metabolism, tissue exposure, dose, safety or botanical exclusivity, and enrichment does not establish therapeutic efficacy. Input provenance and the enrichment calculation are detailed in Methods in S1 File.

### 2.7 Molecular docking and reference-ligand controls

Compound–target prioritization considered recurrence of the compound among at least two of the three highest-ranked herbs, target membership in the DEG–disease intersection, CTD binding or protein-activity evidence, and an experimental structure with a reference ligand and the required cofactors or metals. Primary candidates were drawn from the top-30 PPI hubs; a candidate outside that set was assessed separately as a secondary exploratory extension. Candidate-level evidence and decisions are reported in Table O in S1 Data.

Local docking used native-ligand-defined pockets. The PPARG production receptor was the continuous ligand-binding domain of 1FM6 chain D (residues 207–477), with BRL/rosiglitazone as reference ligand; 2PRG and 7AWC were evaluated as alternative structures. NQO1 used the 2F1O A/C biological dimer with both FAD cofactors and dicoumarol as reference. MMP2 used 1HOV model 1 with Zn/Ca and SC-74020 as reference. Native ligands and waters were removed from receptor files, required cofactors/metals were retained, and protein hydrogens were added with PyMOL 3.1.0. Ligands were prepared with RDKit 2025.09.6 and Meeko 0.8.0. Structure-specific selections, repairs and protonation assumptions are documented in Table I in S1 Data and Methods in S1 File.

Each docking box enclosed the native ligand with 6 Å padding. AutoDock Vina 1.2.7 used exhaustiveness 32, nine modes, an energy range of 5 kcal/mol, and three random seeds (20260904–20260906). MMP2 was tested with AutoDock4Zn maps generated using AutoGrid 4.2.7.x [30–33]. PPARG was also evaluated with Vinardo [34]. Reference-pose recovery required symmetry-corrected heavy-atom RMSD ≤2 Å for the top-ranked pose in at least two of three runs and, for MMP2, plausible Zn coordination. Values of 2–3 Å prompted sensitivity testing; values >3 Å or failure to recover the pocket precluded interpretation under that setting. Query docking used the retained setting. Scores were reported as computational benchmarks rather than measured affinities.

### 2.8 Molecular dynamics and trajectory analysis

Each PPARG–BRL, PPARG–kaempferol, NQO1–dicoumarol and NQO1–taxifolin complex was simulated in three independently seeded 100 ns replicas. GROMACS 2026.3 used Amber14SB protein parameters, TIP3P water and GAFF2/AM1-BCC small-molecule parameters generated with ACPYPE 2026.9.3 [35–40]. PPARG was represented by the isolated 1FM6 ligand-binding domain with neutral ligands; NQO1 contained the A/C dimer, two oxidized FAD^2-^ cofactors and one neutral ligand. Non-protein parameters were checked for missing entries and unjustified zero-force terms. FAD preparation and parameter provenance are described in Methods in S1 File and Table K in S1 Data.

Systems were placed in dodecahedral boxes with 1.0 nm solute clearance, neutralizing ions and 0.15 M NaCl. Steepest-descent minimization was followed by 100 ps restrained NVT and 500 ps restrained NPT equilibration at 300 K. NPT used C-rescale pressure coupling; reference-coordinate scaling was com for PPARG and all for NQO1. Unrestrained production used a 2 fs step, PME electrostatics, hydrogen-bond constraints, V-rescale temperature coupling and Parrinello–Rahman pressure coupling. Completion was verified from target and checkpoint steps, trajectory time spans, energy records and logs. Even 100 ns replicas may miss slow rearrangements; enhanced sampling was not used.

MDAnalysis 2.10.0 and archived scripts were used for structural analysis at 100 ps intervals [41]. Molecules were made whole and placed in consistent periodic images before fitting protein backbone atoms to the unminimized starting structure. The common pocket comprised protein residues within 6 Å of the native reference-ligand heavy atoms. Protein/pocket and protein-fitted ligand RMSD, 4.5 Å heavy-atom contacts, and direct N/O hydrogen bonds were analyzed separately for each replica. Hydrogen bonds required donor–acceptor distance ≤3.5 Å and donor–hydrogen–acceptor angle ≥150°, with a 3.0 Å sensitivity analysis. Water-mediated bridges were not included. PPARG helix-12 residues 466–474 were assessed after fitting the remaining core, with axis orientation, intrasegment backbone hydrogen bonds and ligand contacts as additional measures. The two NQO1 FAD molecules were analyzed separately by whole-cofactor, ring and regional RMSD and linkage torsions.

Operational retention required a ligand centroid within 10 Å of the current common-pocket centroid and at least one pocket-residue contact. A persistent escape required both distance >10 Å and loss of pocket contacts for at least 5 ns. Energy eligibility required at least 90% retained frames in the fixed 50–100 ns window, no persistent escape there, and late-block mean ligand/pocket RMSD changes no greater than 2/1 Å; NQO1 additionally required FAD-ring changes no greater than 1 Å. At least two control and two query replicas had to qualify for a within-target comparison. Results were also examined in 25 ns blocks and at 8/12 Å distance thresholds. These screens detect gross instability and do not demonstrate thermodynamic convergence.

Exploratory MM-GBSA and MM-PBSA estimates were calculated with AmberTools 24.8 MMPBSA.py using a single-trajectory protocol [42]. Each replica contributed 101 snapshots, spaced 0.5 ns apart in the same 50–100 ns window. The NQO1 receptor retained both FAD cofactors; bulk water and ions were removed consistently. Topology conversion preserved MD charges, Lennard–Jones parameters and 1–4 interactions. Calculations used mbondi2 radii, interior/exterior dielectric constants of 1/78.5 and 0.15 M ionic strength. GB used igb = 5; PB used radiopt = 0 and inp = 1. Exact nonpolar and solver settings are in Methods in S1 File and S3 Data. No conformational-entropy correction was included. Per-replica means, between-replica SD, 5 ns block summaries and 1 ns subsampling checks were reported. Trajectory frames were not treated as independent experimental replicates, and values were not interpreted as experimental binding free energies.

### 2.9 Statistical analysis and reproducibility

Statistical analyses used R 4.6.0 and Python 3; package versions and execution records are included in S2 Code. Differential-expression models respected patient pairing, and resampling used complete patient pairs where applicable. Correlation tests were two-sided; over-representation tests were one-sided. Multiplicity correction and significance thresholds are specified for each analysis. Processed expression matrices, sample manifests, enrichment results and numerical figure data are provided in S1 Data, with analysis scripts and protocols in S2 Code and Methods in S1 File. Third-party input exclusions and access limitations are documented in S1 Data.

### 2.10 Use of artificial intelligence tools

Generative artificial intelligence tools (ChatGPT, OpenAI; GPT-5.5) were used solely to assist with English language editing and minor improvements in clarity of the manuscript text. Numerical outputs were produced using the statistical and molecular-simulation software and archived scripts. The authors are responsible for verifying the analyses and references, interpreting the findings and approving the final manuscript.

## 3. Results

### 3.1 Data integration and identification of differentially expressed genes

**Fig 1:**
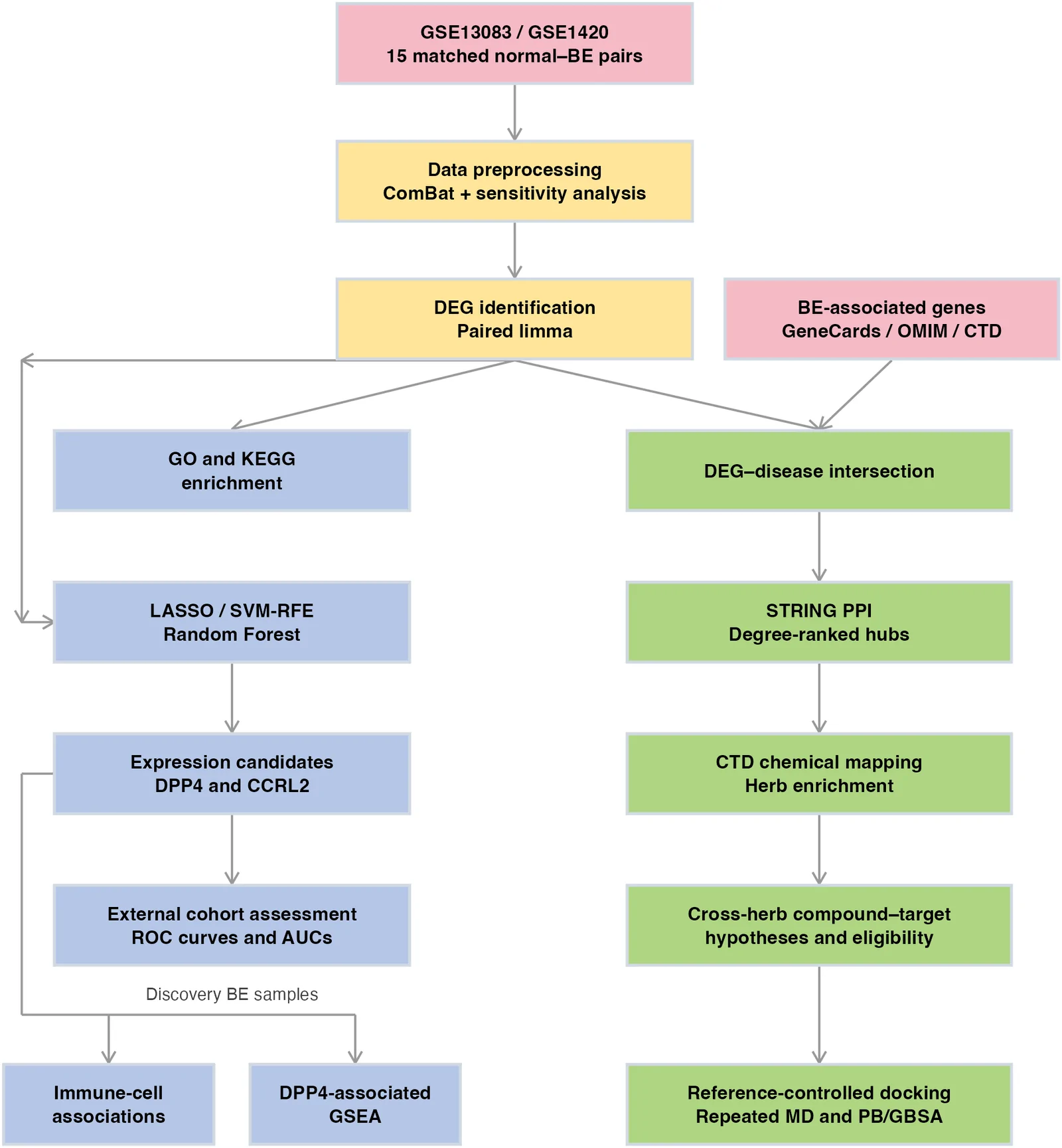
Overall analytical workflow. The transcriptomic and reverse-network branches include paired analysis, expression-candidate assessment, gene-to-herb mapping and reference-controlled structural assessment. DPP4-associated pathway and immune analyses used the discovery BE samples.

**Fig 2:**
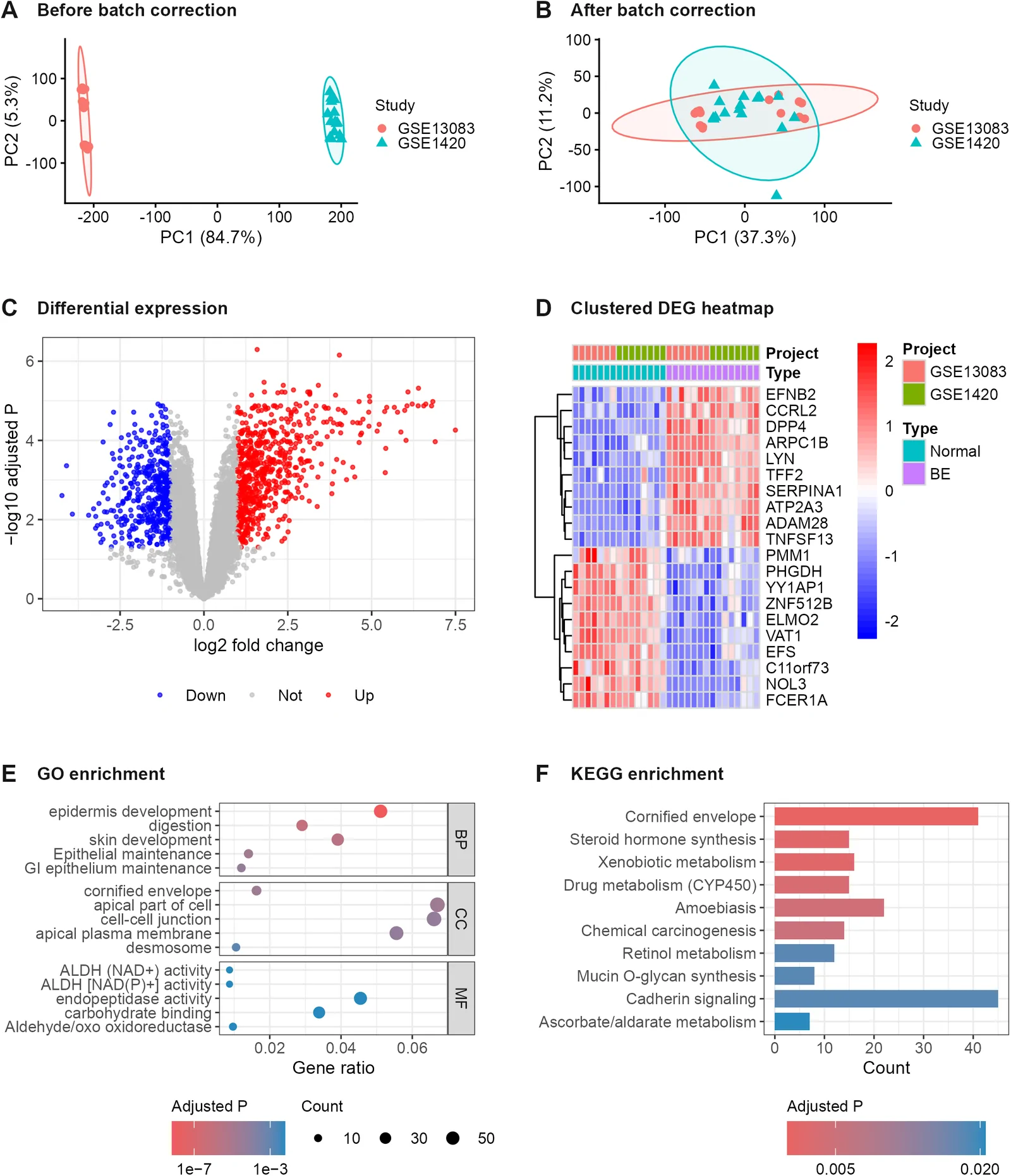
Transcriptomic profiling, batch correction and functional enrichment. (A, B) PCA before and after primary ComBat; color and shape identify GEO cohort (coral circles, GSE13083; teal triangles, GSE1420). Ellipses depict descriptive 95% normal cohort ellipses, not a test of batch removal. (C) Paired-limma volcano plot; red and blue indicate significant up- and downregulated genes at |log2 fold change| > 1 and BH FDR < 0.05. (D) Row-standardized heatmap of the ten upregulated and ten downregulated genes with the lowest FDR in each direction, with hierarchical row clustering, fixed tissue-grouped column order, and study/tissue annotation bars. (E) GO bubble plots show five lowest-FDR terms per ontology; x denotes GeneRatio, size denotes count and color denotes adjusted P. (F) KEGG bars show counts for the ten lowest-FDR pathways; color denotes adjusted P. Enrichment uses the assay-matched background. ALDH denotes aldehyde dehydrogenase. Full term names and displayed data are in S1 Data.

The pair-aware workflow is shown in Fig 1. GSE13083 and GSE1420 formed a 30-sample discovery cohort comprising 15 matched pairs. The primary ComBat matrix contained 13,236 repaired gene-symbol rows. Global cohort-associated partial R^2^ conditional on phenotype decreased from 0.88076 before ComBat to 0.00085 afterward; median gene-level partial R^2^ decreased from 0.90286 to 0.00032; and the variance-weighted partial R^2^ across the first 10 principal components decreased from 0.87737 to 0.00033. The whole-pair permutation P value changed from 0.002 to 1.000 (999 permutations), indicating removal of measured cohort association but not proving preservation of biological signal. Paired limma identified 1,085 DEGs at |log2 fold change| > 1 and BH FDR < 0.05; phenotype-preserving ComBat yielded 1,222 DEGs in sensitivity analysis (Tables H and N in S1 Data; Fig 2).

### 3.2 Functional enrichment analysis of the BE transcriptome

Using assay-matched gene backgrounds, GO analysis identified 121 significant terms and KEGG analysis identified 20 significant pathways (BH FDR < 0.05). Enrichment emphasized epithelial differentiation and maintenance, barrier and cornified-envelope programs, and xenobiotic, steroid and retinol metabolism (Fig 2; S1 Data). These associations do not establish pathway activation or causal effects.

### 3.3 Machine-learning feature-selection results

**Fig 3:**
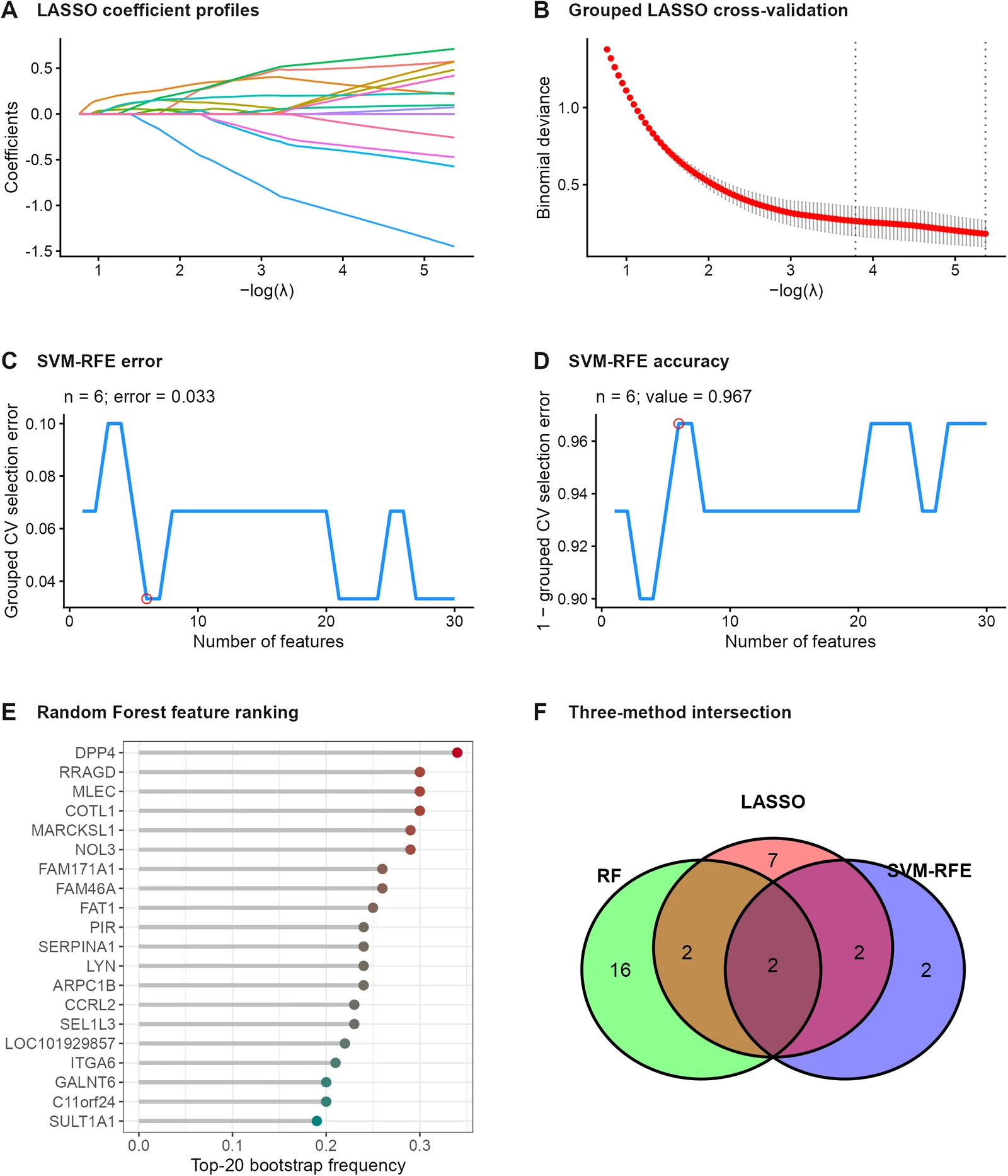
Machine-learning feature selection. (A) LASSO coefficient paths. (B) Five-fold patient-grouped CV deviance; dashed lines mark lambda.min and lambda.1se. (C) SVM-RFE feature-selection error. (D) One minus the error in panel C; the selected feature count is indicated. These are conditional selection diagnostics after full-cohort DEG filtering, not independent predictive validation. (E) Random Forest lollipop plot of top-20 inclusion frequency across 100 complete-pair bootstrap resamples. (F) Three-method Venn diagram; the common set contains DPP4 and CCRL2. Preprocessing-sensitivity results are reported in Table N in S1 Data.

In the primary pair-aware analysis, LASSO retained 13 features, grouped linear SVM-RFE retained 6, and the pair-bootstrap Random Forest stability set contained 20 genes. DPP4 and CCRL2 were the two three-method intersections. DPP4 ranked first in the RF stability ordering and entered the top 20 in 34% of pair bootstraps; CCRL2 ranked 14th and entered in 23%. When ComBat was rerun with phenotype preservation, LASSO retained 13 features, SVM-RFE retained 3, and no feature was shared by all three algorithms. Thus, the primary intersection was preprocessing-sensitive rather than a robust two-gene signature (Fig 3; Tables B, C, and N in S1 Data).

### 3.4 Cohort-specific assessment of DPP4 and CCRL2

**Fig 4:**
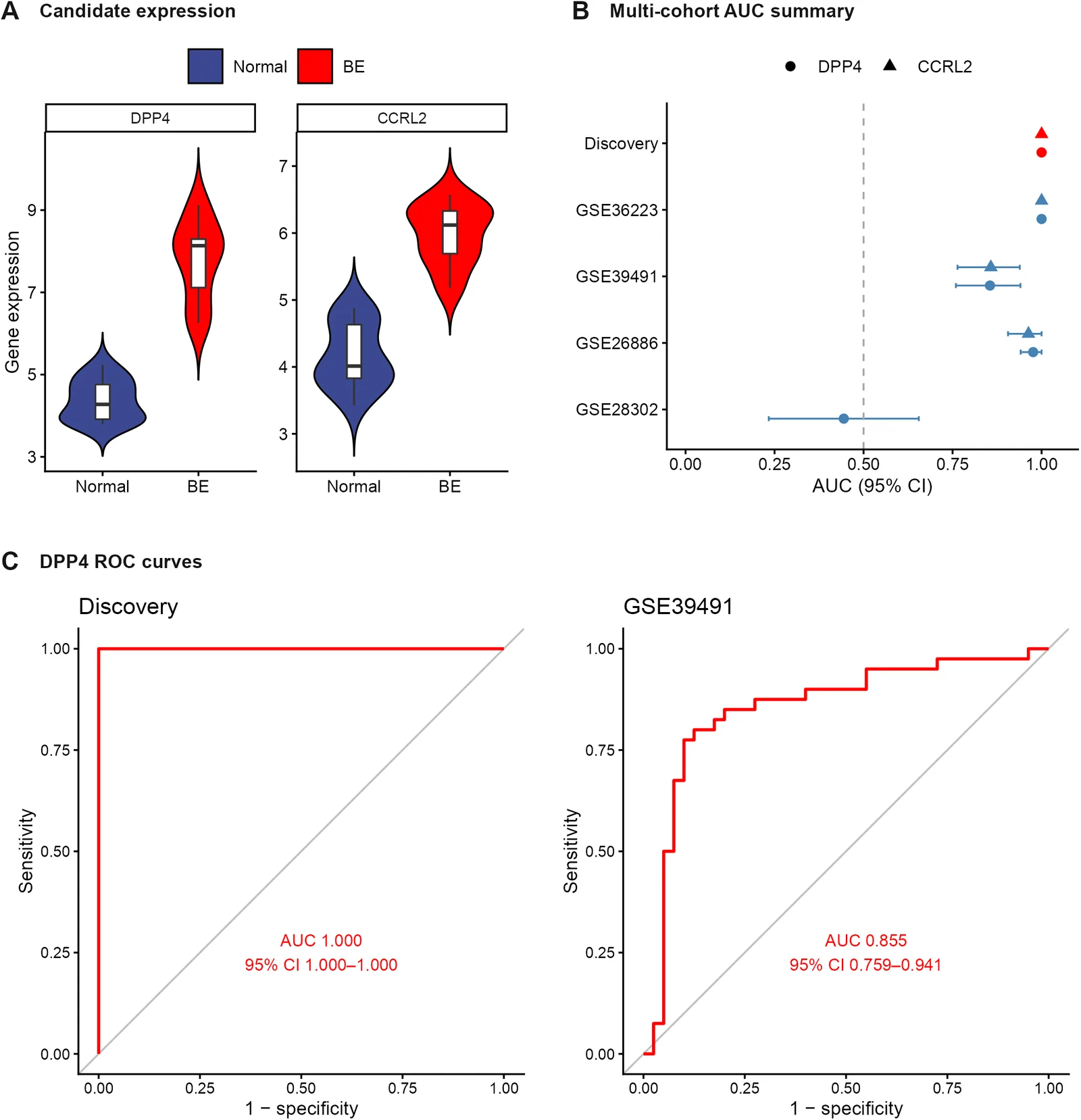
Expression and cohort-specific assessment of DPP4 and CCRL2. (A) Violin/box plots of discovery-cohort expression; boxes show the median and interquartile range, with whiskers extending to values within 1.5 times the interquartile range. (B) AUC forest plot for both genes; circles denote DPP4 and triangles CCRL2, with red discovery and blue external estimates. Confidence intervals use complete-pair bootstrap or DeLong methods according to cohort design. CCRL2 has no uniquely mapped probe in GSE28302 and therefore no estimate there. (C) Red ROC curves for DPP4 in the discovery cohort and GSE39491. ROC direction is fixed; no cohort-specific optimal threshold is displayed. Full cohort and candidate values are provided in Table D in S1 Data.

DPP4 and CCRL2 were both upregulated in the primary paired analysis (DPP4: log2FC = 3.411, FDR = 4.07 × 10^−6^; CCRL2: log2FC = 1.805, FDR = 5.99 × 10^−6^). In GSE36223, both apparent AUCs were 1.000. GSE39491 yielded AUCs of 0.855 for DPP4 (pair-bootstrap 95% CI 0.759–0.941) and 0.858 for CCRL2 (0.764–0.939); GSE26886 yielded 0.976 (DeLong 95% CI 0.941–1.000) and 0.963 (0.906–1.000), respectively. In cross-platform GSE28302, DPP4 yielded a fixed-direction AUC of 0.444 (DeLong 95% CI 0.234–0.655), while CCRL2 had no uniquely mapped GPL2507 probe (Fig 4; Table D in S1 Data).

The AUCs of 1.000 in the discovery cohort and GSE36223 describe complete separation in those samples. Their degenerate empirical bootstrap intervals do not imply zero uncertainty in other populations. DPP4 did not discriminate in the proposed direction in GSE28302 (its confidence interval includes 0.5), and CCRL2 could not be assessed under the specified GPL2507 mapping. These observations do not establish platform-transportable diagnostic performance. No common threshold or calibrated probability model was developed.

### 3.5 Functional enrichment and immune microenvironment profiling of DPP4

**Fig 5:**
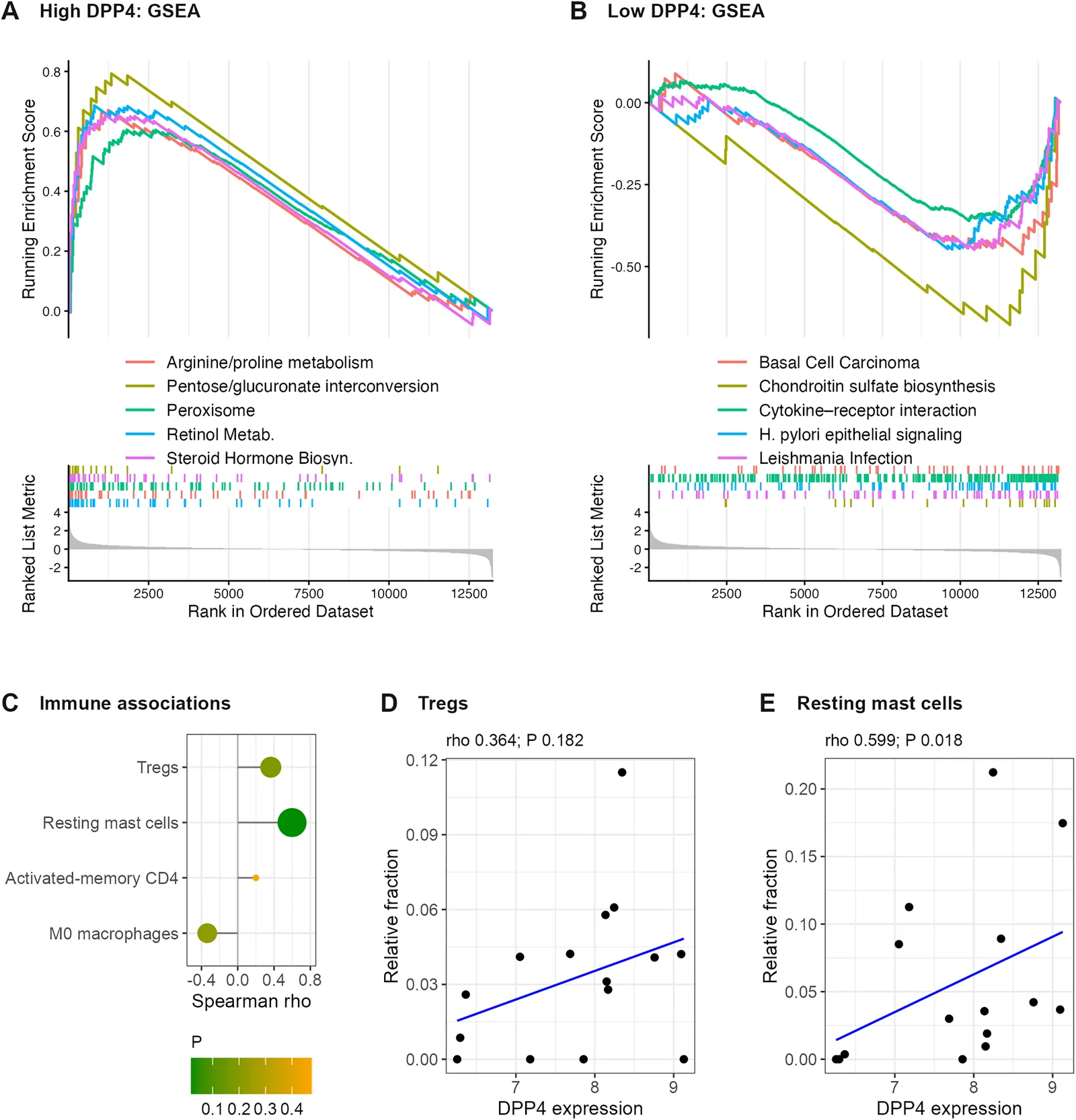
DPP4-associated pathways and immune-cell fractions. (A, B) GSEA running enrichment scores, gene-hit positions and ranked-list metrics for high- and low-DPP4 BE samples. Five terms in each NES direction are selected from the nominal-P < 0.05 output in its ranked order; not all displayed terms have BH FDR < 0.05. Full NES/P/FDR values are in S1 Data. (C) Lollipop summary of the four exploratory simple Spearman associations; size reflects absolute rho and color the nominal P value. (D, E) DPP4 associations with Treg and resting mast-cell fractions in all 15 BE samples. Blue regression lines are visual guides; displayed rho/P values are Spearman statistics. Multiplicity correction and epithelial-adjusted associations are reported in Table E in S1 Data and Fig A in S1 File. None of the four simple associations remained significant after BH correction.

Because DPP4 was the pre-existing biological focus and ranked first in the primary pair-bootstrap RF analysis, its pathway and immune-fraction analyses were retained as exploratory follow-up. CCRL2 remained a parallel expression candidate; this prioritization did not establish DPP4 superiority. Median-split GSEA associated higher DPP4 with retinol, amino-acid, peroxisomal, and lipid-metabolism gene sets, whereas glycosaminoglycan and cytokine/chemokine gene sets showed negative enrichment. GSEA evaluated 186 gene sets, of which 17 had BH FDR < 0.05 (Fig 5). These associations describe transcriptional gene-set differences and not biochemical pathway activation.

In the 15 BE samples, DPP4 showed a positive association with estimated Treg fraction (rho = 0.364, P = 0.182) and a nominal association with resting mast-cell fraction (rho = 0.599, P = 0.018), but neither remained significant after BH correction (FDR = 0.293 and 0.074, respectively). Adjustment for the epithelial-marker composite attenuated both associations (Treg: partial rho = 0.196, P = 0.503; resting mast cells: partial rho = 0.468, P = 0.092; Fig A in S1 File; Table E in S1 Data). These data support only exploratory bulk-tissue compositional associations.

### 3.6 Gene-to-herb reverse network pharmacology

**Fig 6:**
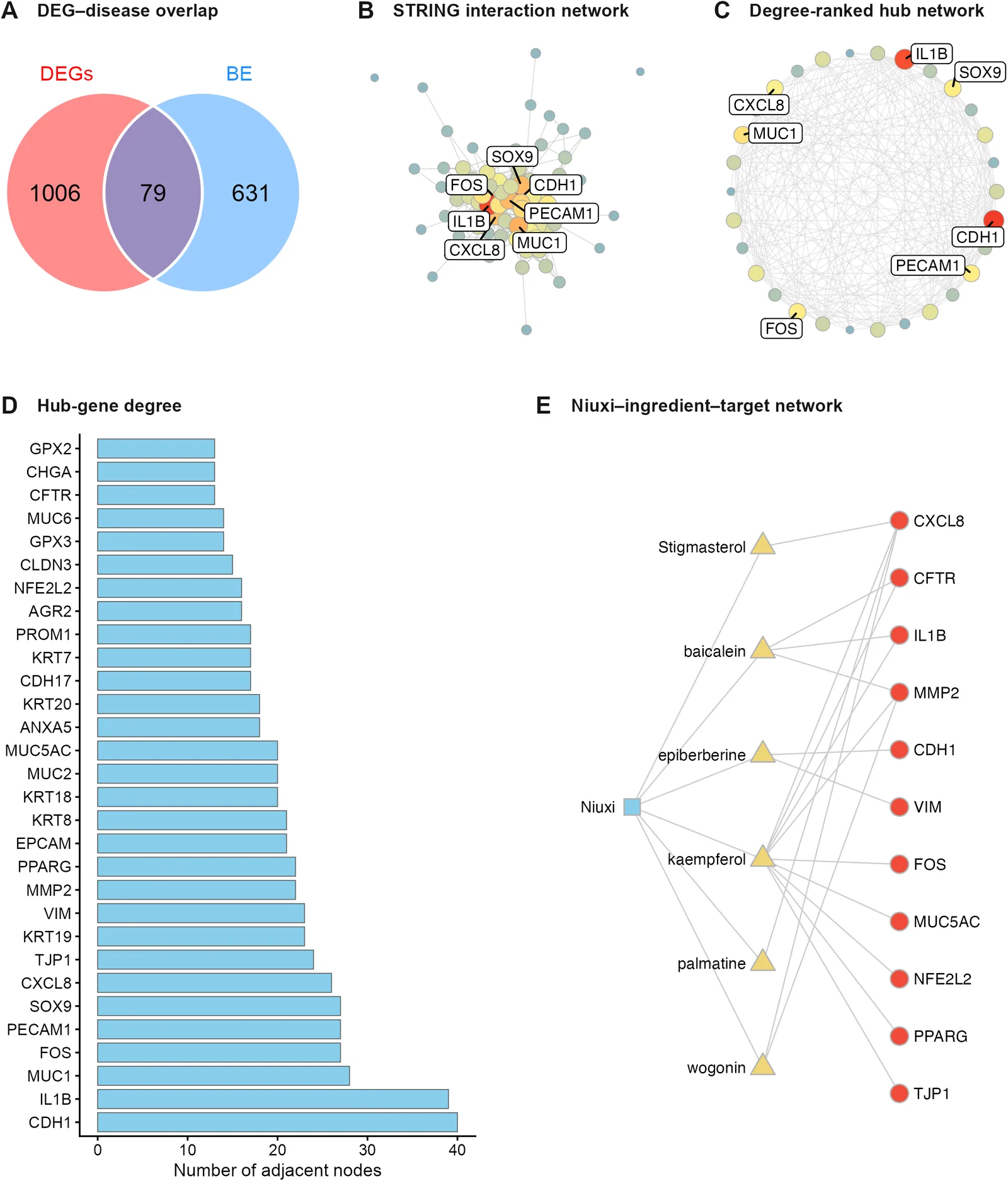
Reverse network pharmacology. (A) Overlap of discovery DEGs and disease-associated genes. (B) STRING network of the 79 submitted genes, including the two isolated vertices; labels identify selected high-degree nodes. (C) Circular layout of the top-30 hub subnetwork. Node size and color in B/C indicate degree, with warmer colors indicating higher degree. (D) Degree bars for the 30 hubs. (E) Niuxi–ingredient–target network: the blue square represents the herb, yellow triangles its mapped ingredients and red circles the associated targets. Edges denote database associations and do not establish regulation, exposure, direct binding or efficacy. Quantitative herb enrichment is shown in Fig F in S1 File; full networks and rankings are in S1 Data. Node degree and database edges are descriptive; no node- or edge-specific P values were calculated.

The 710-gene disease union intersected the 1,085 primary DEGs at 79 genes (Fig 6A). STRING returned 460 unique edges involving 77 genes; HPSE and PLCE1 had no returned edge. CDH1 and IL1B had the highest degrees (40 and 39), while VIM, MMP2, and PPARG ranked 10th, 11th, and 12th (degrees 23, 22, and 22); NQO1 ranked 32nd (degree 13) and was outside the primary top-30 hub set (Fig 6B–6D). CTD filtering of the 30 hubs yielded 24,191 human records and 2,992 unique chemicals; 42 chemicals mapped to the 2,555-ingredient herb universe. Twenty-three of 363 eligible herbs met both nominal and BH-adjusted P < 0.05. Niuxi ranked first (6 overlapping ingredients; fold enrichment 18.25; adjusted P = 1.62 × 10^−4^), Mahuang second (6; 15.87; adjusted P = 2.03 × 10^−4^), and Mahuanggen third (4; 18.72; adjusted P = 0.00491; Fig F in S1 File). Niuxi overlaps were stigmasterol, baicalein, epiberberine, kaempferol, palmatine, and wogonin (Fig 6E; S1 Data). These are database associations, not evidence of constituent exposure or therapeutic activity.

### 3.7 Positive-control docking and query evaluation

Application of the pre-docking eligibility screen assigned kaempferol–PPARG as the primary structural hypothesis on the basis of cross-herb recurrence, PPARG network priority, and CTD binding/activity annotations [43]. Independent microscale-thermophoresis evidence supports kaempferol–PPARG binding and reports context-dependent antagonism [44], whereas taxifolin–NQO1 entered as a secondary hypothesis supported by activity/regulatory rather than direct-binding evidence [45]. VIM and FOS pairs were excluded before query docking, while the MMP2 candidates supported by cellular activity/expression records [46,47] proceeded to metal-aware reference-control testing (Table O in S1 Data). PPARG redocking was receptor- and scoring-dependent. The older 2PRG settings failed. In 7AWC, all 12 evaluated combinations of alternate conformation, scoring function, and seed recovered top-ranked BRL poses within 2 Å; kaempferol was reproducible within each alternate-conformation receptor and across the two receptors. Because 7AWC required internal loop modeling for MD, the complete 1FM6 chain-D receptor was evaluated. Vina was borderline (BRL RMSD 1.966–2.094 Å), whereas the Vinardo sensitivity passed in all three seeds (0.735, 0.762, and 1.880 Å). Kaempferol under the passed 1FM6–Vinardo protocol gave top scores of −5.325 to −5.328 kcal/mol and a median pairwise top-pose RMSD of 0.00284 Å. Its protein-aligned pose differed from the 7AWC pose (ligand RMSD 5.91 Å; centroid separation 3.31 Å), demonstrating that the hypothesis is receptor dependent rather than a unique binding mode. BRL and kaempferol topology gates both passed. With the repaired 2F1O FAD coordinates used for MD, the secondary NQO1 control recovered dicoumarol at 0.439–0.446 Å, and taxifolin top poses remained reproducible (scores −8.943 to −8.968 kcal/mol; median pairwise RMSD 0.024 Å). Original-versus-repaired receptor top-pose RMSDs were 0.042 Å for dicoumarol and 0.020 Å for taxifolin; the coordinate repair did not change the docking decision, and the zero-amplitude N8–C22 series was traced to the explicit X–c3–na–X term in the official GAFF2 2.2.30 file. Parameter-file identity and format conversion were verified; this provenance check was not a FAD-specific quantum-mechanical validation. The final AutoDock4Zn MMP2 control gave Zn contacts of 1.989–2.056 Å but top-pose RMSDs of 3.526–3.602 Å, so MMP2 queries did not enter MD (Figs B, C, and D in S1 File; Tables F, I, J, K, and O in S1 Data).

### 3.8 Molecular dynamics analysis

All 12 replicas reached 100 ns and passed the runtime integrity checks. Both PPARG ligands maintained contacts with the native-pocket region throughout the fixed 50–100 ns window under the primary 10 Å definition. The protein-fitted ligand RMSD was 1.37 ± 0.47 Å for rosiglitazone and 6.10 ± 0.37 Å for kaempferol (mean of three replica means ± between-replica SD), indicating closer preservation of the reference pose and reorientation of kaempferol within the receptor-associated region. Core-fitted helix-12 RMSD was 2.00 ± 0.36 and 2.44 ± 1.14 Å, respectively, with heterogeneous orientation and contact patterns across replicas (Fig E in S1 File; Table G in S1 Data). At the stricter 8 Å threshold, retention in kaempferol replicas 2 and 3 was 81.2% and 5.0%, respectively, compared with 100% under the primary definition. Thus, the observed retention reflects continuing pocket-region association rather than preservation of a single pose.

In the secondary NQO1 comparison, dicoumarol and taxifolin maintained pocket-region contacts in all three replicas. The corresponding ligand RMSD was 1.66 ± 0.54 Å for dicoumarol and 4.93 ± 1.52 Å for taxifolin, indicating greater query-pose displacement. Taxifolin replica 2 retained 100% of fixed-window frames at 10 Å and 80.4% at 8 Å. The FAD isoalloxazine rings remained comparatively localized, while late rearrangements in some cofactors were concentrated in the adenine/ribose portion (Figs G and H in S1 File; Table G in S1 Data). Together, these observations identify receptor-associated taxifolin configurations that merit direct-binding evaluation.

Both endpoint models yielded negative estimates for the reference and query complexes. All replicas met the recorded operational energy criteria and were included in the same fixed window. For PPARG, MM-GBSA estimates were −42.11 ± 1.56 kcal/mol for rosiglitazone and −30.22 ± 5.82 kcal/mol for kaempferol; MM-PBSA estimates were −30.13 ± 1.12 and −24.92 ± 7.23 kcal/mol, respectively. For NQO1, MM-GBSA estimates were −32.77 ± 4.24 and −19.96 ± 3.55 kcal/mol for dicoumarol and taxifolin, with MM-PBSA estimates of −28.87 ± 3.41 and −15.59 ± 2.80 kcal/mol. These are entropy-uncorrected endpoint estimates with between-replica SD, not measured affinities. Full components, block summaries and subsampling checks are provided in S3 Data (Fig I in S1 File). Both models favored the reference ligands at the group level. Kaempferol showed greater between-replica variation, with individual PB estimates overlapping those of rosiglitazone. The endpoint results complement the sustained pocket contacts by characterizing the energetics of the receptor-associated configurations sampled by each compound.

## 4. Discussion

DPP4 and CCRL2 were shared by the three primary feature screens, but the intersection was absent under phenotype-preserving ComBat. Their strong discrimination in three Affymetrix cohorts did not translate into cross-platform validation. The expression results therefore prioritize exploratory, preprocessing-sensitive candidates rather than a validated biomarker panel.

Three methodological contributions structure this study. First, complementary LASSO, pair-grouped SVM-RFE, and pair-bootstrap Random Forest screens were integrated under a patient-pair-aware design, while preprocessing sensitivity was used to expose rather than conceal feature instability. Second, a disease-gene-to-STRING-hub-to-CTD-chemical-to-herb workflow prioritized candidate herbs without prespecifying a botanical intervention, while preserving database provenance and directionality limits. Third, reference-ligand controls and repeated MD separated pocket-region retention from preservation of a static docking pose. The query poses rearranged despite continuing receptor contacts, and the trajectories exposed helix-12 or cofactor heterogeneity that a single docked image could not capture. These contributions support hypothesis prioritization, not experimental validation of diagnostic or therapeutic utility.

DPP4 remained the highest-ranked primary pair-bootstrap RF feature, but its 34% top-20 frequency and loss from the phenotype-preserved LASSO/SVM-RFE intersection argue against describing it as a stable consensus biomarker. CCRL2 was also a primary three-method intersection, but it lacked a uniquely mapped GPL2507 probe. The appropriate next step is prospective, assay-standardized evaluation of both candidates rather than construction of a diagnostic rule from these retrospective arrays.

DPP4 (CD26) is a membrane ectopeptidase and signaling protein expressed in epithelial and immune compartments [48–50]. A small immunohistochemical study of 12 surgical specimens detected DPPIV in Barrett columnar non-goblet cells and associated neoplastic cells but not in goblet cells, providing direct evidence that a tissue-level DPP4 signal can accompany an enterocyte-like non-goblet phenotype [51]. Single-cell studies resolve distinct Barrett epithelial states and immune contamination [52,53], but they do not justify assigning the present bulk-array signal to a specific compartment. Acid and bile salts can induce epithelial-mesenchymal plasticity in human Barrett organoids [54], providing a reflux-injury context rather than evidence that DPP4 drives that response. Immune-cell CD26 could separately reflect T-cell abundance or activation and modify peptide/chemokine signaling. These alternatives require spatial protein validation and perturbation experiments.

Consistent with this compartmental distinction, DPP4 associations with Treg and resting mast-cell fractions weakened after epithelial-marker adjustment and did not survive multiplicity correction. CIBERSORT relative fractions cannot quantify absolute immune infiltration, and bulk tissue cannot distinguish epithelial DPP4 from immune-cell CD26.

The 79-gene network contained epithelial/barrier nodes (CDH1, MUC1, TJP1 and keratins), inflammatory nodes (IL1B and CXCL8), and remodeling/metabolic nodes (VIM, MMP2 and PPARG), consistent with heterogeneous epithelial injury and metaplastic remodeling rather than one causal pathway [55–60]. The top-30 cutoff was operational rather than biological: NQO1 ranked 32nd, and small changes in the paired DEG intersection altered hub membership, the CTD chemical pool, and herb order. This sensitivity reinforces that degree and enrichment ranks prioritize hypotheses but do not measure causal importance.

Niuxi, Mahuang, and Mahuanggen ranked first through third, respectively, but traditional-use descriptions, modern preclinical pharmacology, and the enrichment statistic represent different evidence levels. Niuxi is the root of *Achyranthes bidentata* and is traditionally described as invigorating blood, unblocking channels, and strengthening sinews and bones; none of these categories is a recognized treatment rationale for BE [17]. Broad experimental reports of anti-inflammatory, antioxidant, and wound-healing activities offer only a hypothesis-level connection to reflux-associated mucosal injury and remodeling and do not demonstrate prevention of esophageal fibrosis or intestinal metaplasia. The six Niuxi overlaps—stigmasterol, baicalein, epiberberine, kaempferol, palmatine, and wogonin—are not unique to Niuxi [61,62]. Mahuanggen is the Ephedra root/rhizome and is pharmacognostically distinct from the Mahuang stem. Database mapping does not establish plant content, extraction yield, oral exposure, dose, safety, or efficacy.

The structure-selection rule separated biological relevance from technical tractability. Kaempferol–PPARG had the strongest primary network/CTD rationale, and complete 1FM6 supported a matched BRL-control/kaempferol-query MD test after a scoring-function sensitivity step. The different kaempferol poses in 1FM6 and 7AWC preclude a unique-pose claim and make the MD findings specific to the initialized 1FM6 state. In contrast, the open, solvent-exposed, Zn-dependent MMP2 cleft did not recover the reference inhibitor with the final metal-aware method and did not enter MD. VIM and FOS were excluded because indirect CTD actions and the absence of validated drug-like pockets or controls could not support direct-binding tests. The FAD-containing NQO1 dimer recovered dicoumarol and supported a reproducible taxifolin pose, but NQO1 ranked 32 rather than within the top 30, the source study reported activity/regulation rather than direct binding, and the FAD model remains a generic fixed-state approximation despite verified parameter provenance. NQO1 therefore remains a secondary exploratory analysis and cannot validate the primary network or herb ranking. Both PPARG and NQO1 were upregulated in BE, but expression direction alone does not define a beneficial intervention. Kaempferol has published PPARG-binding and antagonism evidence [44], whereas taxifolin-associated NQO1 activity may arise through ARE-mediated regulation [45]. BRL and dicoumarol are structural reference ligands; their pharmacological actions need not match those of the query compounds. The workflow did not test expression reversal or efficacy in BE. Repeated MD showed sustained pocket-region contacts after query-pose rearrangement, and both endpoint models yielded negative query-complex estimates. These findings provide structural and energetic support for prioritizing kaempferol–PPARG for experimental testing; taxifolin–NQO1 remains a secondary direct-binding hypothesis. Helix-12 and FAD variation defines the conformational scope of these results; receptor activation and enzyme modulation require functional assays.

Several limitations materially constrain interpretation. The discovery cohort contained only 15 matched pairs. Full-cohort ComBat and DEG filtering preceded resampling, and model selection used the available CV results; the feature-selection curves are therefore potentially optimistic and are not end-to-end nested validation. Although pairing and grouped resampling were corrected, the primary feature intersection was not reproduced after phenotype-preserving ComBat, and Random Forest top-20 frequencies were low. External datasets used different depositor processing; three Affymetrix cohorts produced strong separation, whereas the Illumina DPP4 AUC was 0.444 with a confidence interval including 0.5 and CCRL2 could not be uniquely mapped there. Clinical covariates including dysplasia grade, proton-pump-inhibitor exposure, *Helicobacter pylori* status, age, and reflux severity were unavailable or inconsistent. Bulk tissue cannot identify the cellular source of DPP4 or CCRL2, and no prospective threshold or protein assay was evaluated. The top-30 cutoff was operational, chemical matching was exact-name based, CTD actions were context specific, and enrichment cannot establish botanical content, plasma or esophageal exposure, direction of therapeutic effect, dose, toxicity, or efficacy. The PPARG query pose was receptor/scoring dependent; production uses an isolated 1FM6 ligand-binding domain without RXR or coactivator peptide and fixed neutral ligand microstates. MMP2 failed its metal-aware control, and VIM/FOS lacked defensible direct-binding models. NQO1 remains secondary; repaired-FAD sensitivity reproduced the original docking poses, its generic FAD parameter provenance was verified, although a FAD-specific potential-energy validation was not performed. Twelve 100 ns trajectories do not exhaust slow conformational transitions. The 10 Å pocket rule is operational, and boundary sensitivity and late helix/cofactor rearrangements limit claims of pose stability or convergence. Single-trajectory MM-PBSA/GBSA estimates omit conformational entropy and unbound-state reorganization, depend on dielectric/radius and fixed-protonation assumptions, and are not experimental binding free energies. The 100 ps structural sampling does not resolve fast hydrogen-bond kinetics, and water-mediated interactions were not evaluated. No new SPR, ITC, enzyme, cell, organoid, animal, pharmacokinetic, or metabolite experiments were performed in this study. Historical database-search dates and GeneCards score cutoffs were unavailable. CIBERSORT was run without permutations, so deconvolution quality was not screened using sample-level permutation P values.

## 5. Conclusion

The integrative analysis prioritized DPP4 and CCRL2 as exploratory, preprocessing-sensitive expression candidates and Niuxi as the highest-ranked database-derived herb hypothesis. Reference-controlled docking and repeated MD provided structural and energetic support for testing kaempferol–PPARG and the secondary taxifolin–NQO1 extension. The simulations identified sustained pocket-region contacts with query-pose reorientation. Prospective tissue, protein, exposure, binding and functional studies are required before diagnostic or therapeutic interpretation.

## Supporting information

S1 File Supplementary methods and figures. Detailed computational procedures, input and structure preparation, MD and endpoint-analysis methods, and supplementary Figs A-I with legends.
(PDF)

S1 Data Analytical inputs, supplementary tables and numerical results. Tables A-O are indexed in TABLE_INDEX.md; original CSV filenames are retained for code compatibility. The archive includes processed expression matrices, cohort manifests, enrichment and reverse-network outputs, docking inputs and metrics, and the herb–ingredient table supplied as raw input.
(ZIP)

S2 Code Analysis and figure-generation scripts, run order, software-session records, and structure-preparation and trajectory/endpoint-analysis code. (ZIP)

S3 Data MD model inputs, per-replica structural and endpoint data, parameter-conversion and quality records, analysis scripts and coordinate-reconstruction instructions. An identical copy accompanies the analyzed trajectories in the Figshare record identified in Data availability.
(ZIP)
