# Supplementary material for: Integrative transcriptomics and reverse network pharmacology prioritize DPP4 and Achyranthes bidentata in Barrett’s esophagus
Source: PLoS One. 2026 Sep 25;21(9):e0359324. doi: 10.1371/journal.pone.0359324 (PMC13614539; doi:10.1371/journal.pone.0359324)
Supplement: Supplementary file 1 [file pone.0359324.s001.pdf]

S1 File

Supplementary methods and figures

Integrative transcriptomics and reverse network pharmacology prioritize DPP4 and *Achyranthes bidentata* in Barrett's esophagus

This file contains detailed computational methods and Figs A-I. Tables A-O are supplied in S1 Data; analysis code is in S2 Code and MD model and analysis files are in S3 Data.

1. Expression matrices and sample identity

Discovery files are merge.preNorm_symbol_repaired.txt and merge.normalize_symbol_repaired.txt in S1 Data/transcriptomics. The sample manifest links every sample to its GEO accession, tissue condition and patient pair. Probe-to-symbol mapping and averaging preceded matrix merging; spreadsheet-converted symbols were repaired using GPL96. Clinical covariates not present in the original records were not imputed. External cohorts were kept separate. The primary and phenotype-preserving ComBat matrices are distinct analysis specifications, with the same subsequent feature-screening algorithm.

2. Batch diagnostics

For each expression matrix, fit the reduced model with phenotype and the full model with phenotype plus study. Compute partial R^2 from residual sums of squares; for the global statistic, sum across genes before dividing. PCA uses centered, unscaled expression. For the permutation diagnostic, shuffle the seven/eight study labels across 15 whole patient pairs using seed 20260906, and map the permuted label to both tissue observations. Use 999 permutations and the add-one empirical P-value formula. The fixed-matrix comparison is descriptive: it is not a permutation of an entire preprocessing workflow, nor proof that biological effects have been preserved. Full assignment and statistic tables accompany Table H in S1 Data.

3. GO/KEGG mapping and background

Use all expression rows included in differential testing. Map exact SYMBOL to ENTREZID with org.Hs.eg.db, then query ALIAS only for unmapped symbols. Retain only symbols mapping to one unique Entrez identifier; deduplicate identifiers. Apply identical mapping to the DEG foreground. GO uses enrichGO(ont="ALL", pool=FALSE), with BH correction within each ontology. KEGG uses archived human TERM2GENE and TERM2NAME snapshots dated 2026-09-04. Both use minGSSize=10, maxGSSize=500 and explicit universe=background. The effective denominator is the subset annotated in each database, so it can differ from the submitted background size. All tested terms and mapping exclusions are archived.

4. Machine-learning implementation

LASSO uses family="binomial", alpha=1, type.measure="deviance", standardized predictors, five patient-grouped folds, seed 20260904 and lambda.min. SVM-RFE uses linear SVM weights squared, cost=10 during ranking, and repeated removal of half the features above 50 and one thereafter. Outer-fold training rankings are averaged for final ordering; inner four-fold CV tunes cost over 2^(−6:6), and the first minimum in the 1–30 feature sweep determines set size. These inner folds do not rerun the outer-training feature ranking. RF uses seeds 300001–300100 and complete-pair bootstrap sampling, with 1,000 trees per forest. Full-cohort preprocessing and DEG filtering precede resampling; these curves and stability frequencies are conditional exploratory diagnostics, not unbiased end-to-end validation.

5. CIBERSORT and immune associations

The recovered invocation is CIBERSORT("ref.txt", "merge.normalize.txt", perm=0), using the local v1.03 implementation whose QN default is TRUE. Its max(Y)<50 check triggers conversion by 2^Y before quantile normalization. LM22 contains 547 signature genes, of which 530 match the mixture. All 30 samples are retained without a permutation P-value filter; only the 15 BE samples enter the reported correlations. The full archived fraction matrix was reproduced using these settings. Supply a licensed CIBERSORT implementation and LM22 reference to the verification wrapper in S2 Code; input checksums and the comparison output are in S1 Data. The package does not assert redistribution rights over the CIBERSORT implementation or reference signature. Four immune-cell types are evaluated as exploratory follow-up; partial Spearman uses ranked-variable residualization, Pearson residual correlation and approximate t inference with $n-3$ degrees of freedom.

6. Reverse network pharmacology

Disease gene lists are the archived GeneCards, OMIM and CTD exports. Their historical database queries, dates and GeneCards score thresholds are not recoverable; the privately retained source lists and their published file hashes define the analyzed input. Raw exports and their union are excluded from the public copy. The herb–ingredient table is provided as the raw input in S1 Data/network_inputs/refer.Drug.csv. Its columns are Chinese_name, Drug and Ingredient. Query STRING with species=9606, required_score=400 and no added interactors. Rank nodes by unique-edge degree. Extract Homo sapiens CTD chemical–gene records for the top 30 genes. For herb enrichment, N is the number of unique ingredients in the herb map, n is the number of retrieved chemicals matching that map, K is the size of an eligible herb ingredient set and k is its overlap with n. Calculate $Pr(X \geq k)$ using the hypergeometric distribution and BH-adjust all eligible herb sets, including zero-overlap sets. Herb set eligibility is $5 \leq K \leq 500$. Ranking and associations do not imply target causality, constituent exposure or reversal of disease expression.

7. Structure preparation and local docking

PPARG: use 1FM6 chain D residues 207–477, remove incomplete terminal Pro206, RXR/coactivator and other chains, waters and the native BRL from the receptor file; retain BRL separately as the reference pose. No internal residues are modeled. 7AWC and 2PRG are alternative docking structures; 7AWC has unresolved residues 264–269 and incomplete Leu270 and is not the production MD construct. NQO1: use 2F1O chains A/C with both FAD cofactors, remove native dicoumarol and waters from the receptor. Rebuild the deposited near-coincident FAD O2A coordinate using local phosphate geometry and the corresponding bond length in human NQO1 structure 8OK0; repeat reference/query docking on the repaired receptor. MMP2: use 1HOV model 1, retain two Zn and two Ca ions and use native SC-74020 as reference. Exact receptor and ligand files, configurations, atom-mapped pose metrics and scores are supplied in S1 Data. Inspect the highest-ranked reference pose for each seed using symmetry-corrected heavy-atom RMSD in the receptor coordinate frame; do not fit the ligand independently. Passing redocking calibrates pose recovery for the known reference; it does not establish query binding or reliable affinity ranking.

8. MD preparation and parameter provenance

PPARG used isolated 1FM6 chain D residues 207–477 with one rosiglitazone or kaempferol molecule. NQO1 used the 2F1O A/C dimer, two oxidized FAD^{2-} cofactors and one dicoumarol or taxifolin molecule. Ligands were modeled in the neutral microstates used in preparation. This fixed-state model does not establish the dominant solution or bound-state microstate. Protein parameters were Amber14SB, water TIP3P and non-protein components GAFF2/AM1-BCC. The FAD atom named N8 in the generated topology corresponds to native N10 and C22 to native C1′. Its zero-amplitude X–c3–na–X term was traced to the official GAFF2 2.2.30 parameter file. The complete local parameter-file SHA-256 matched the official distribution, and conversion retained the zero-energy terms and all 1–4 pairs. Zero amplitude was not treated as missing parameterization, and no FAD-specific torsion fit or quantum-mechanical accuracy validation was performed.

Each complex had three independently seeded 100 ns production trajectories. PPARG NVT velocity seeds were 202609047–202609052; NQO1 seeds were 202609041–202609046. Dodecahedral boxes had 1.0

nm solute clearance, neutralizing ions and nominal 0.15 M NaCl. Steepest-descent minimization used emtol=1000 kJ mol^{-1} nm^{-1}. Restrained equilibration comprised 100 ps NVT and 500 ps NPT at 300 K. C-rescale pressure coupling used refcoord-scaling=com for PPARG and all for NQO1. Production was unrestrained with 2 fs steps, PME electrostatics, 1.0 nm real-space electrostatic and van der Waals cutoffs, EnerPres dispersion correction, hydrogen-bond constraints, V-rescale temperature coupling and isotropic Parrinello–Rahman coupling at 1 bar. Original coordinate and energy output intervals were 10 ps. The separate 100 ps benchmark was not appended to production; production began from the NPT checkpoint.

9. Structural post-processing and operational criteria

Runtime completion was checked using TPR targets, CPT step/time, full trajectory spans and energy/log inspection, not completion markers alone. Basic screens assessed finite energies, absence of serious constraint warnings, minimized maximum force, mean tail temperatures within 300±10 K, and plausible aqueous-system density. These screens are not tests of equilibrium. Post-processing rules were recorded during revision before inspection of the structural and endpoint outputs; they were not an originally preregistered protocol.

Structural analysis sampled 0–100 ns every 100 ps. GROMACS made each molecule whole. PPARG ligand images were chosen relative to the current native pocket; for NQO1, chain A anchored the chain-C image, each FAD was placed relative to its starting protein neighborhood, and the ligand was imaged relative to the shared pocket. The whole protein backbone was fitted to the unminimized initial complex. Native reference-ligand protein contacts within 6 Å defined the same pocket-residue set for reference and query. Pocket RMSD used the corresponding backbone atoms; ligand RMSD used fixed heavy-atom identities after protein fitting, without independently fitting the ligand. First-frame RMSD sensitivity is also supplied.

Residue contacts required any heavy-atom pair within 4.5 Å. Direct hydrogen bonds required an N/O donor with explicitly bonded H, a chemically defined N/O acceptor, donor–acceptor distance ≤ 3.5 Å and D–H–A angle ≥ 150°; 3.0 Å sensitivity is supplied. Water bridges, sulfur/C–H bonds and hydrogen-bond lifetimes were outside this definition. All time series, residue/bond occupancies and 25 ns blocks are provided. Circular means were used for linkage torsions.

PPARG helix 12 residues 466–474 were specified from the chain-D HELIX record in 1FM6. Its backbone RMSD and centroid/axis changes were calculated after fitting core backbone residues 207–465. Intrasegment i-to-i+4 backbone hydrogen bonds and ligand/Tyr473 contacts provide additional geometry. The absence of RXR and coactivator peptides limits functional interpretation. NQO1 FAD molecules were evaluated separately by protein-fitted whole-cofactor and ring RMSD, internally fitted ring RMSD, ring planarity, terminal-ring angle, regional adenine/ribose/ribityl/diphosphate RMSD and the C20–N8–C22–C23 torsion in generated atom names. Whole-cofactor rearrangement was distinguished from loss of the isoalloxazine binding platform.

A retained frame required ligand-centroid distance ≤ 10 Å from the current pocket heavy-atom centroid and at least one 4.5 Å contact with a native-pocket residue. Persistent escape required both distance>10 Å and absence of pocket contacts continuously for ≥ 5 ns. Eligibility in the fixed 50–100 ns endpoint window required ≥ 90% retained frames and no persistent escape. The difference between 50–75 and 75–100 ns mean ligand/pocket RMSD had to be ≤ 2/1 Å; NQO1 FAD-ring means additionally had to differ by ≤ 1 Å. At least two reference and two query replicas were required for a within-target comparison. Helix 12 motion was an outcome, not a forced agonist-like eligibility condition. Contact patterns and cofactor/helix changes were visually examined. Distance sensitivity at 8 and 12 Å was reported. Operational eligibility does not establish an unchanged pose or thermodynamic convergence, and no favorable subwindow or replica was substituted after viewing energy results.

10. Endpoint energy calculations

AmberTools 24.8 MMPBSA.py used a single-trajectory scheme. Protein was the PPARG receptor; protein plus both FAD cofactors was the NQO1 receptor. Water and bulk ions were removed consistently. ParmEd 4.3.1 converted the actual GROMACS parameters, with checks of atom order, charges, Lennard–Jones terms, bonds/angles and all 1–4 pairs/scales. Zero-amplitude periodic terms encoded with period 0 were written with formal period 2 for Amber serialization; their energy and force remained zero. This was not parameter refitting.

Each replica contributed 101 uniformly spaced snapshots from 50 to 100 ns at 0.5 ns intervals. Parallel chunks covered frames 1–51 and 52–101 without overlap and were merged by source-frame identity. GB used OBC2/igb=5, mbondi2 radii, interior/exterior dielectric 1/78.5 and saltcon=0.15 M. GB nonpolar solvation used LCPO area with coefficient 0.0072 kcal mol^{-1} $Å^{-2}$ and zero offset. PB used the same radii, radiopt=0, interior/exterior dielectric 1/78.5, ionic strength 0.15 M (150 mM in generated Sander input), inp=1, surface coefficient 0.00542 and offset 0.92, probe radius 1.4 Å, grid spacing 0.5 Å, fillratio=4 and up to 1000 solver iterations. Actual input/output files accompany S3 Data.

Conformational entropy and separate unbound-state reorganization were not calculated. Endpoint totals therefore must not be interpreted as experimental standard binding free energies or converted to Kd. Each replica was summarized separately, followed by mean and SD across three independent replica means. No frame-based standard error or independent-frame significance test was used. Five-nanosecond block means and 1 ns subsampling checks characterize temporal sensitivity, not proof of complete sampling. Results were compared only within the same target.

11. Data reconstruction

S3 Data contains model files, numerical results, parameter-conversion audits and reconstruction instructions. The analyzed solute coordinates are organized in the Figshare draft record with reserved DOI 10.6084/m9.figshare.33511891. Repository files S4_Data.zip–S9_Data.zip contain the six PPARG trajectories at 100 ps sampling; S10_Data.zip–S21_Data.zip contain the six NQO1 trajectories in nonoverlapping 0–49.9 and 50–100 ns parts. Reference coordinates accompany each part, and both FAD cofactors are retained for NQO1. These subsets reproduce the reported structural analysis and fixed-window energy snapshots; full solvent trajectories are not included. The analysis scripts and computational environments are documented in S2 Code and S3 Data. Runtime source-file hashes are retained as provenance.

12. Third-party input access and public-file integrity

S1 Data/THIRD_PARTY_INPUTS.json lists the original hashes of nine excluded files, including raw disease exports and their union, CTD input records, KEGG snapshots, the KEGG_LEGACY GMT and its full-gene-set RDS duplicate. The herb–ingredient table is provided as the raw input and documented in RAW_INPUT_README.txt. THIRD_PARTY_NOTICES.txt gives provider links and the scope of the rights check. Exact historical retrieval of the excluded third-party inputs remains unconfirmed. Current database versions must not be substituted and presented as exact reproduction. S2 Code/check_external_inputs.py checks identity after authorized inputs are obtained; it does not obtain permission. Retained-output checks do not establish a complete independent rerun.

Personal paths, user identifiers and hostnames in textual runtime records were replaced with neutral labels. Parameters, coordinates and numerical results were unchanged. Original-source hashes continue to identify private originals; MANIFEST.sha256 identifies public files. Fig 5 running-score and hit-track values are retained as study-derived figure data without the full gene-set collection.

Fig A DPP4–immune-fraction correlations

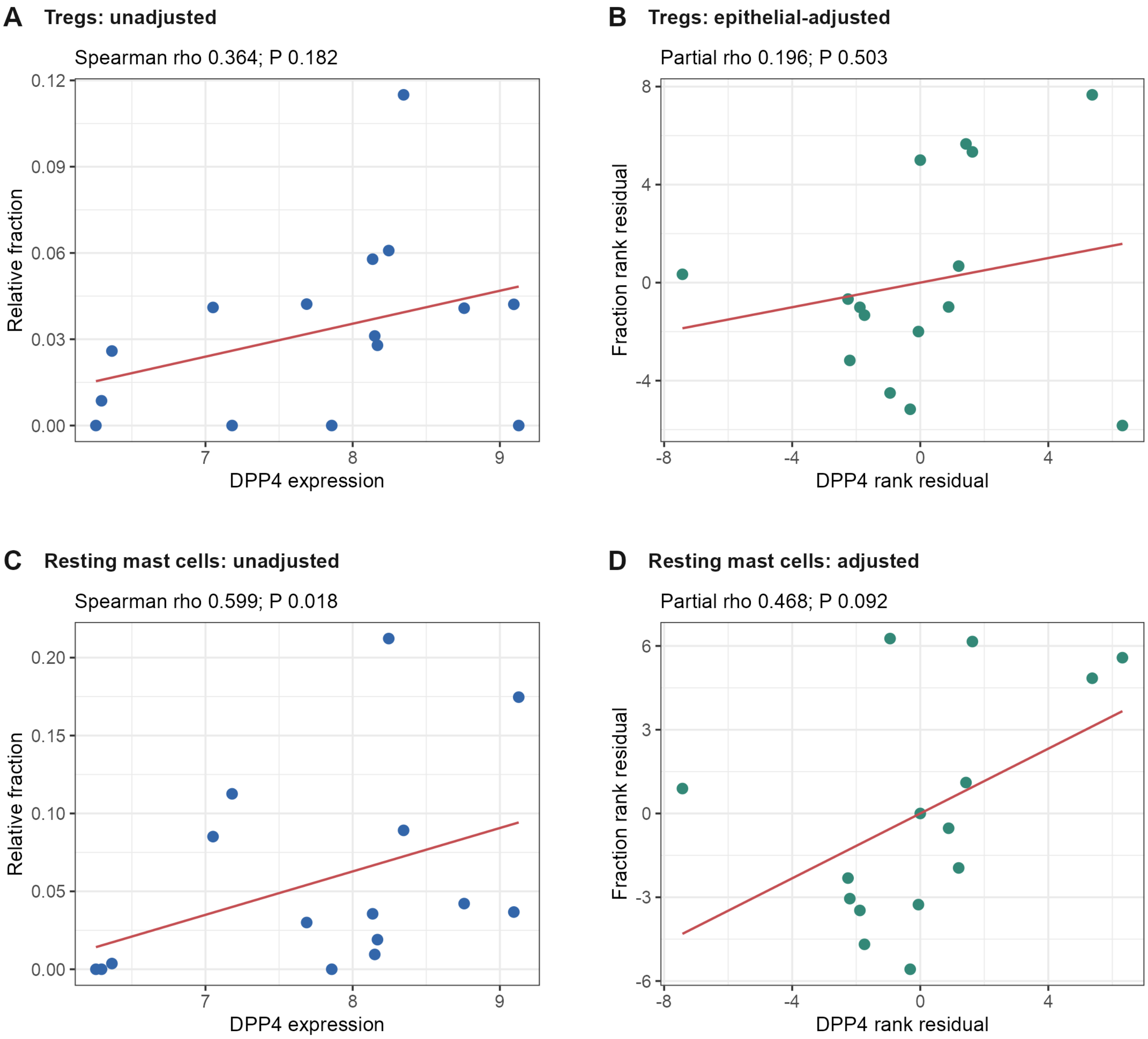

Fig A. DPP4–immune-fraction correlations. Simple Spearman and rank-based partial Spearman associations with regulatory T-cell and resting mast-cell relative fractions in the 15 BE samples. Adjusted panels show rank residuals after regression on the ranked epithelial-expression score; straight lines are visual guides. Approximate adjusted P values use n − 3 degrees of freedom.

Fig B Reference-control pose recovery

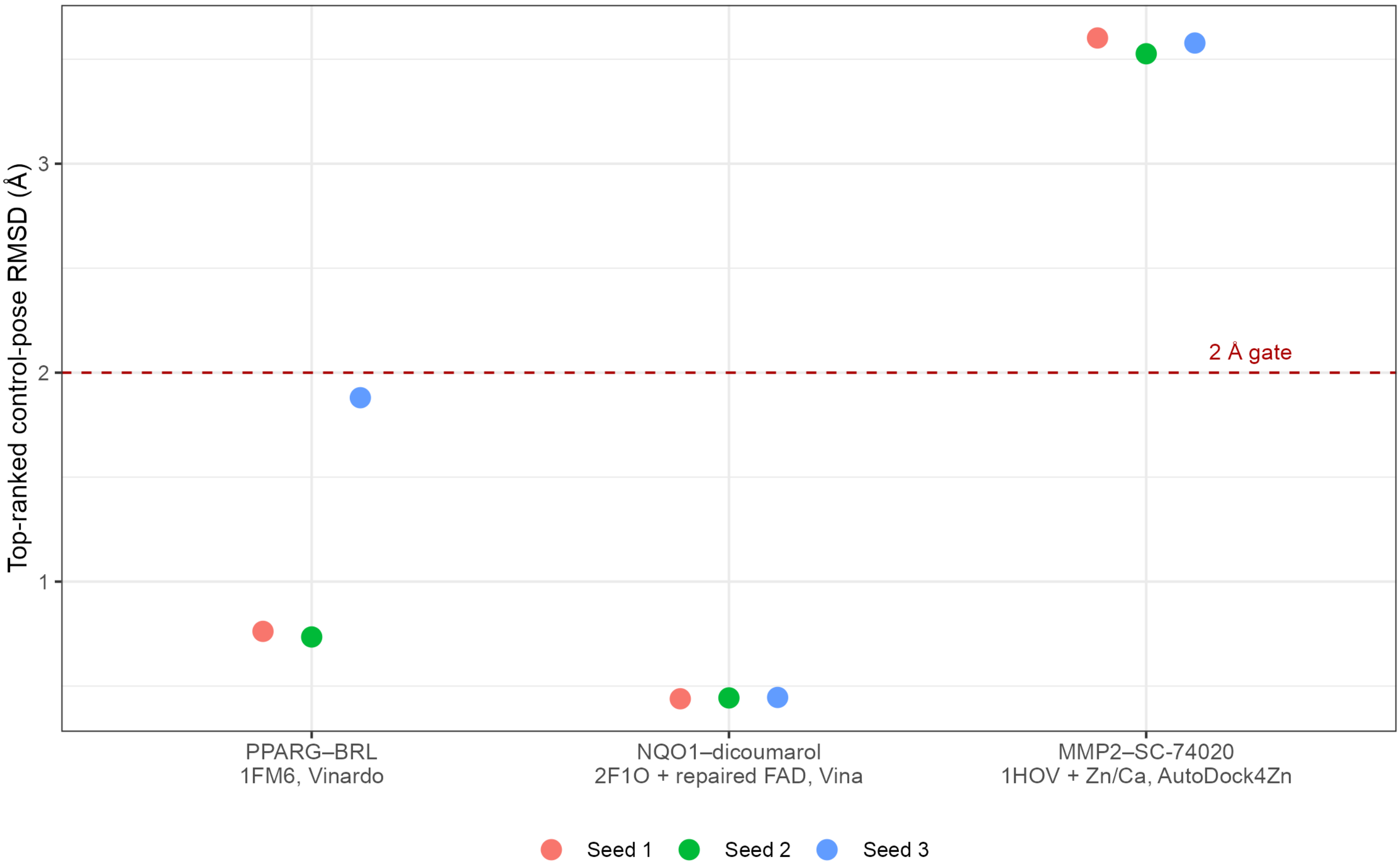

Fig B. Reference-control pose recovery. The dashed line marks the 2 Å top-ranked-pose gate. The displayed production-receptor setting is PPARG–BRL in 1FM6 with Vinardo; NQO1–dicoumarol passed after applying the FAD coordinate repair used for MD; and the final metal-aware MMP2 control failed. The 2PRG, 7AWC, original-FAD NQO1, and other sensitivities are reported in Table J in S1 Data.

Fig C Repaired-FAD NQO1 control and query poses

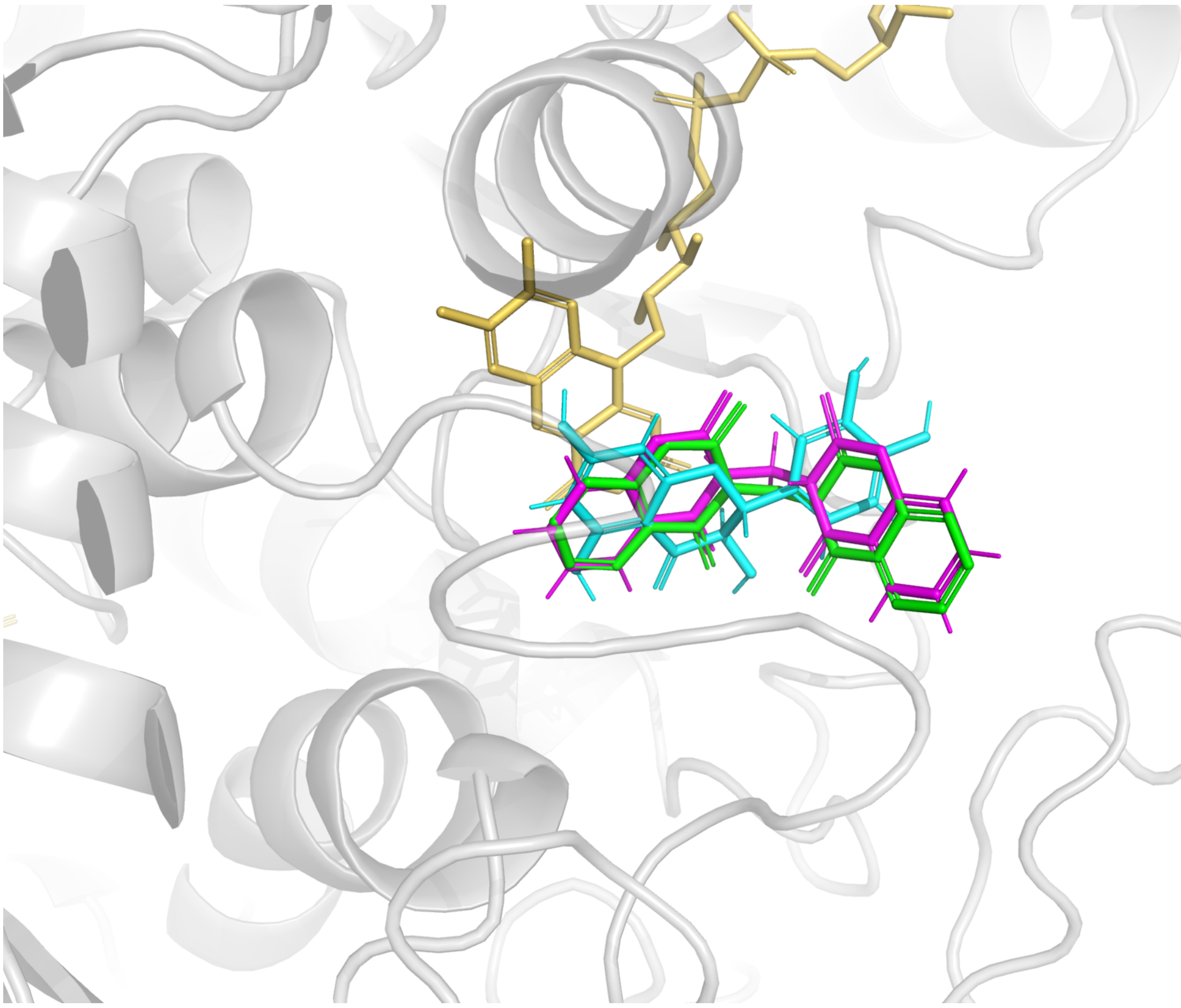

Fig C. Repaired-FAD NQO1 control and query poses. Native dicoumarol (green), a representative redocked dicoumarol top pose (magenta), and a representative taxifolin top pose (cyan) are shown in the 2F1O A/C dimer pocket with both repaired FAD cofactors retained (yellow). NQO1 is a secondary exploratory analysis outside the primary top-30 network; docking does not establish direct taxifolin binding.

Fig D PPARG control and query poses in the complete 1FM6 production receptor

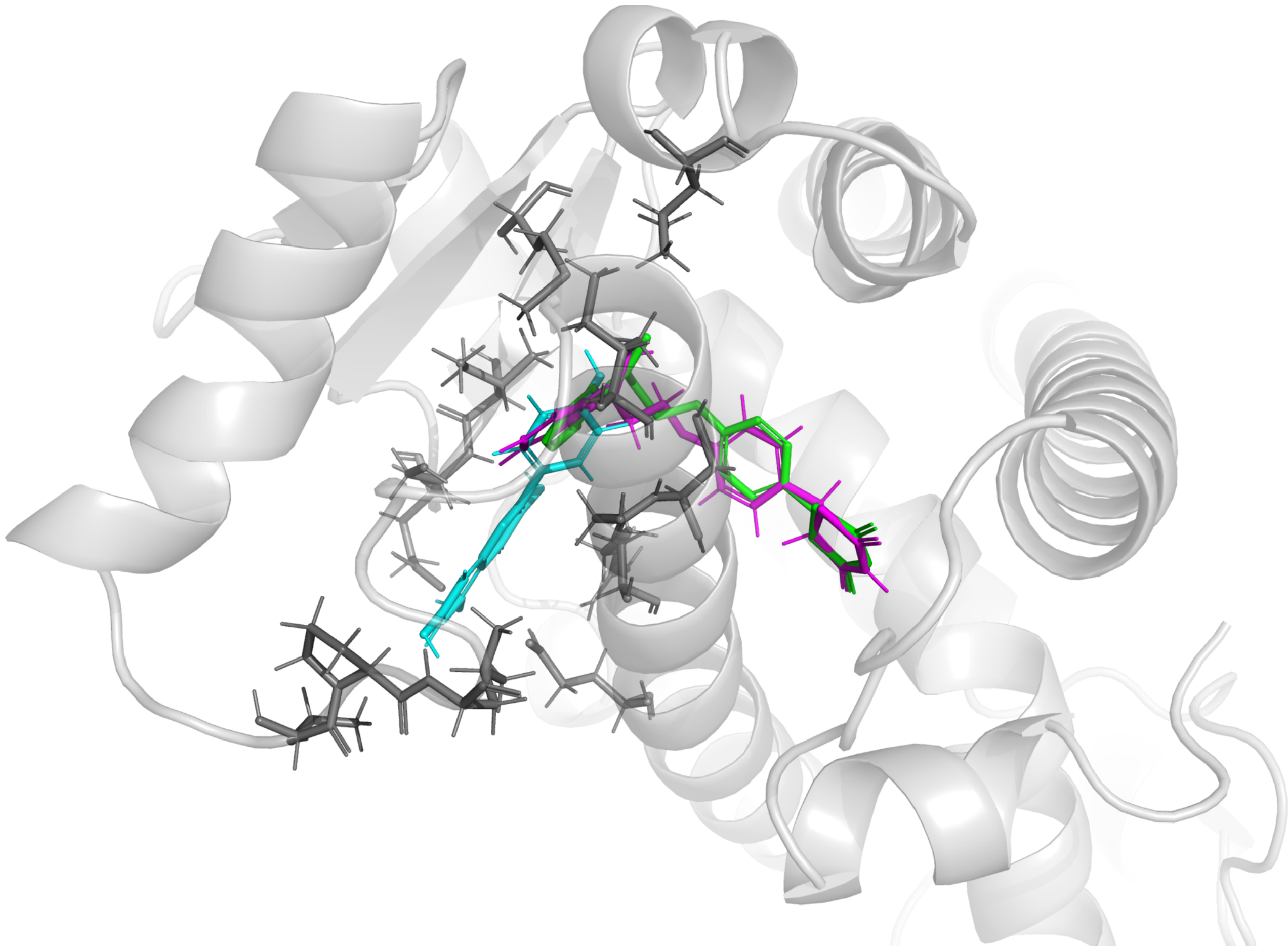

Fig D. PPARG control and query poses in the complete 1FM6 production receptor. Native BRL/rosiglitazone (green), a representative Vinardo-redocked BRL top pose (magenta), and the representative kaempferol top pose (cyan) are shown with nearby pocket residues. The 7AWC docking sensitivity produced a different kaempferol pose and is tabulated separately.

Fig E PPARG replicate MD analyses

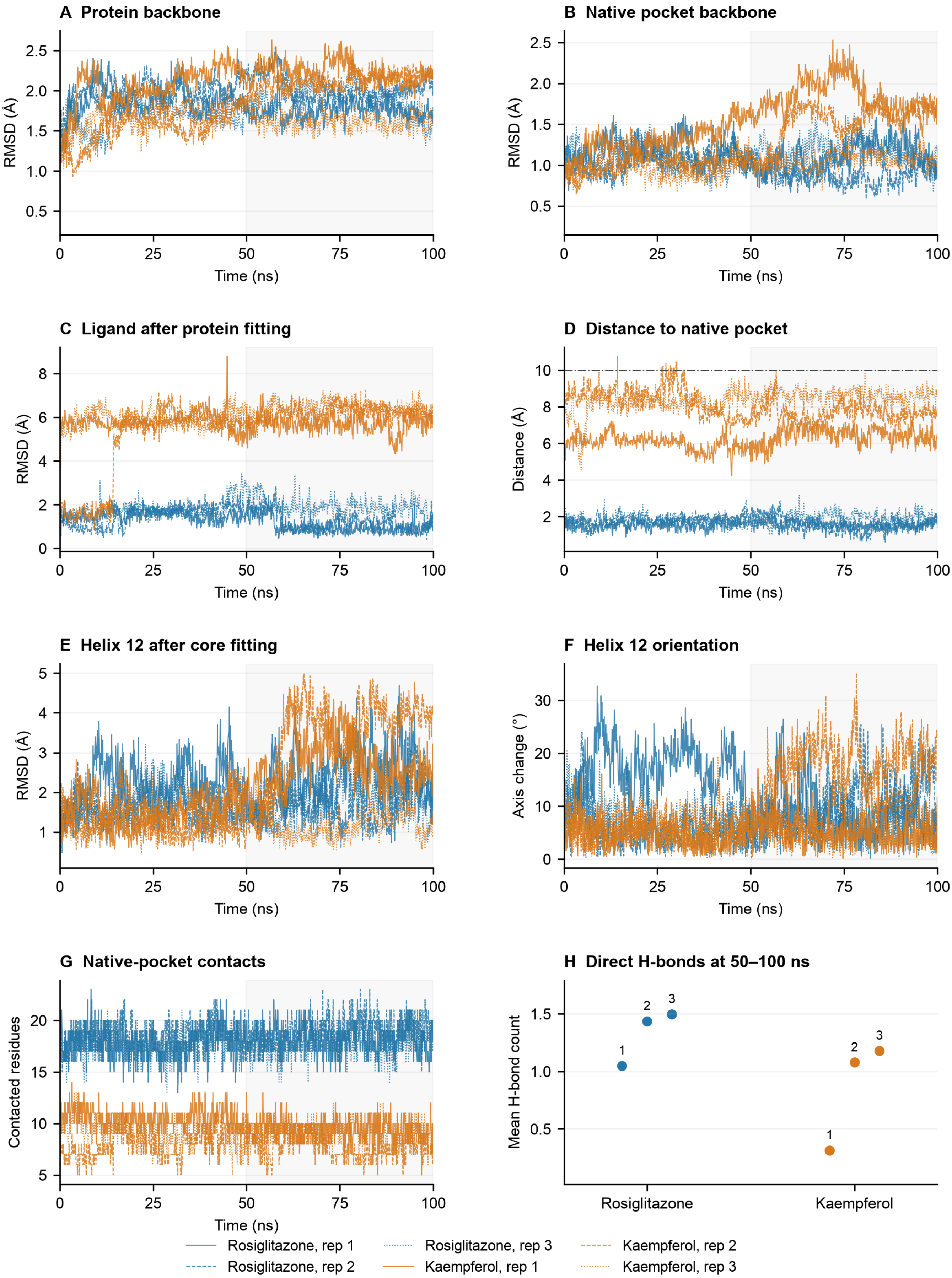

Fig E. PPARG replicate MD analyses. Protein/pocket and protein-fitted ligand RMSD, pocket distance, core-fitted helix-12 RMSD and orientation, pocket contacts and direct hydrogen-bond counts are shown for all three reference/query replicas. Helix 12 comprises residues 466–474. Curves use 100 ps sampling without smoothing; shading identifies the fixed 50–100 ns analysis window, not proven equilibrium. Pocket-distance and hydrogen-bond criteria are defined in Methods in S1 File.

Fig F Quantitative herb enrichment

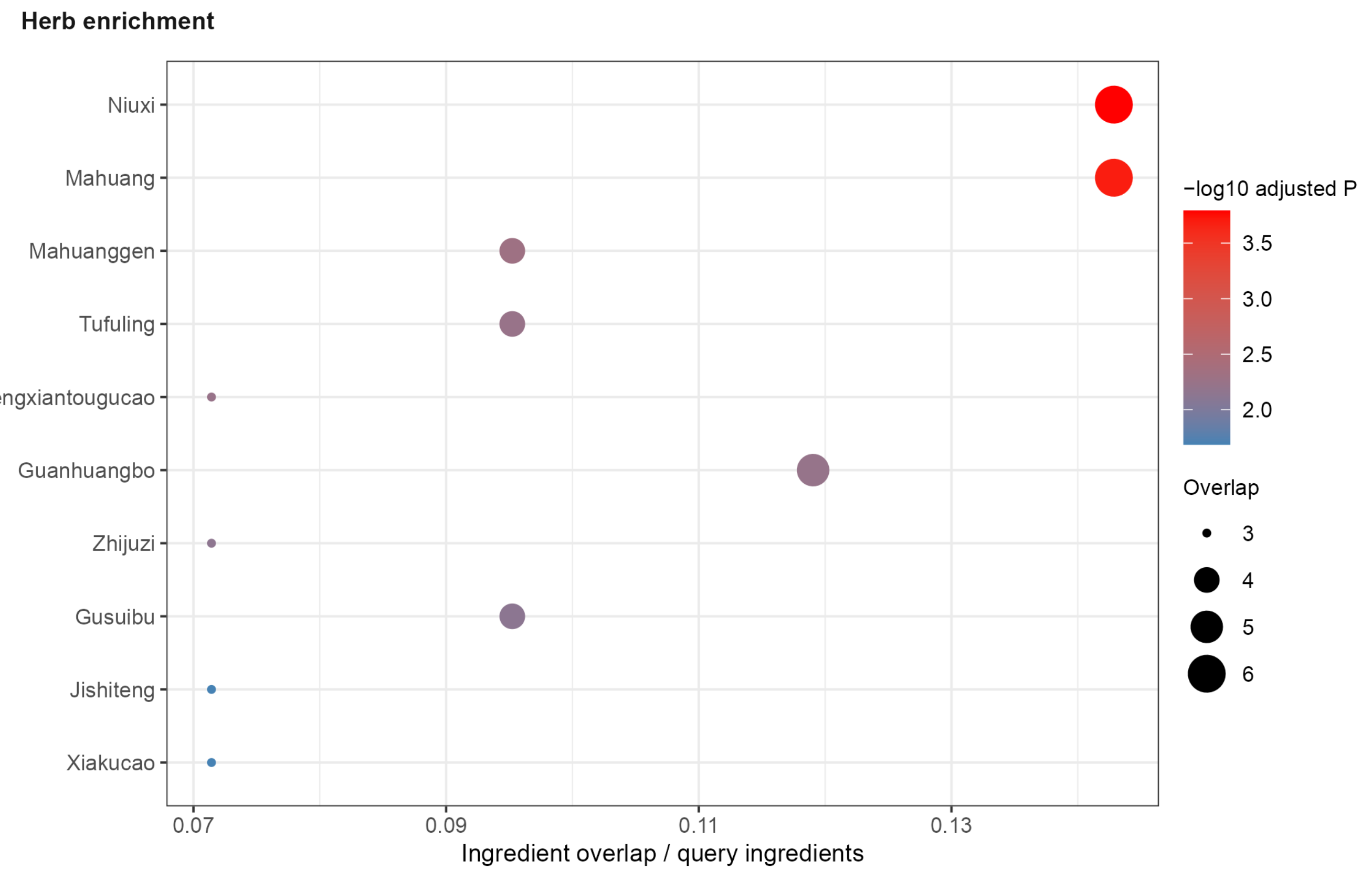

Fig F. Quantitative herb enrichment. The ten lowest-adjusted-P herbs are shown with ingredient overlap divided by the query-ingredient count on the x-axis, overlap as point size and −log10 BH-adjusted P as color. This complements the networks in Fig 6.

Fig G NQO1 replicate MD analyses

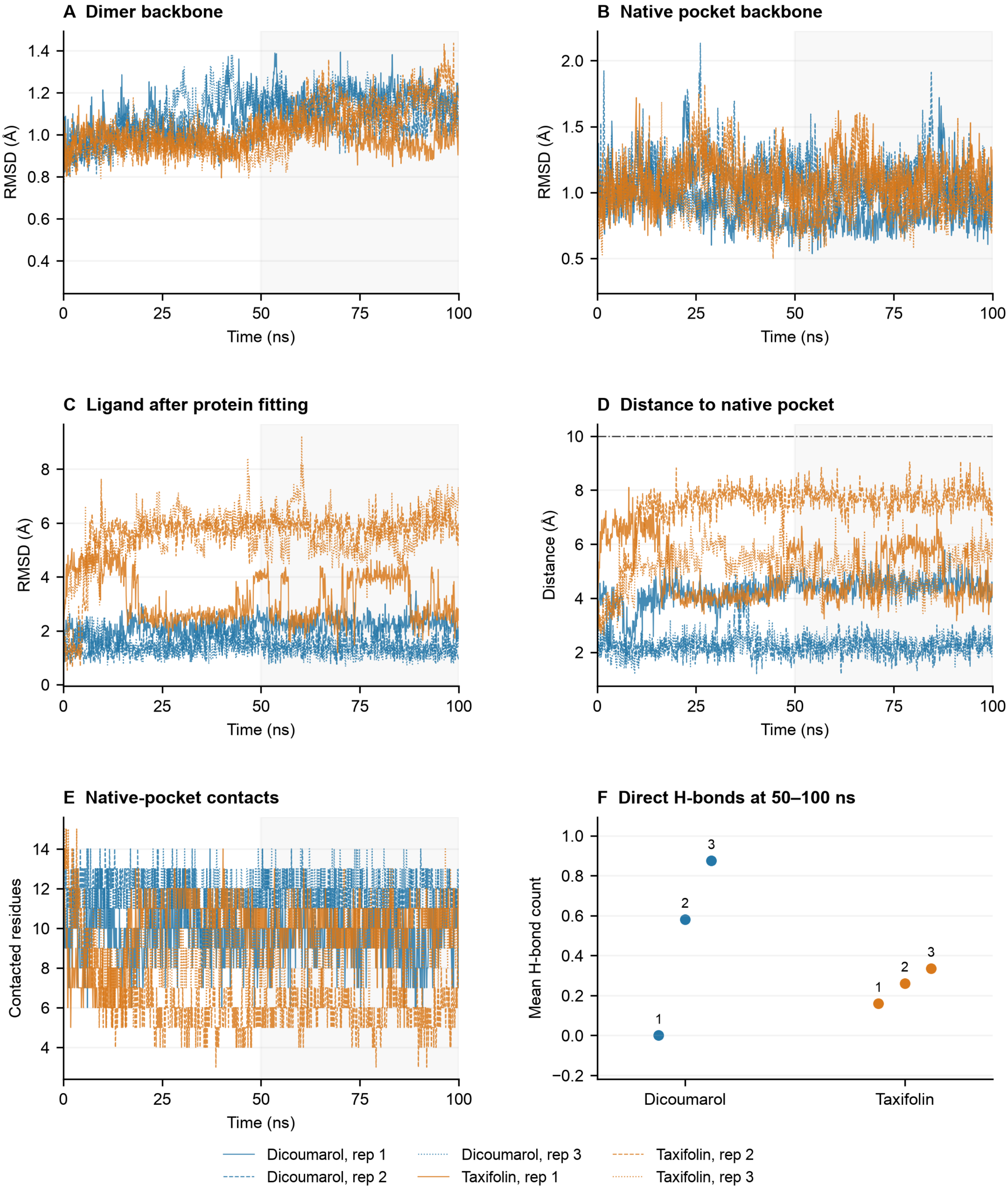

Fig G. NQO1 replicate MD analyses. Dimer/pocket and protein-fitted ligand RMSD, pocket distance, contacts and direct hydrogen-bond counts for three dicoumarol and three taxifolin replicas. Hydrogen-bond points are independent-replica means; curves use 100 ps sampling. Shading indicates the fixed 50–100 ns window.

Fig H NQO1 FAD behavior

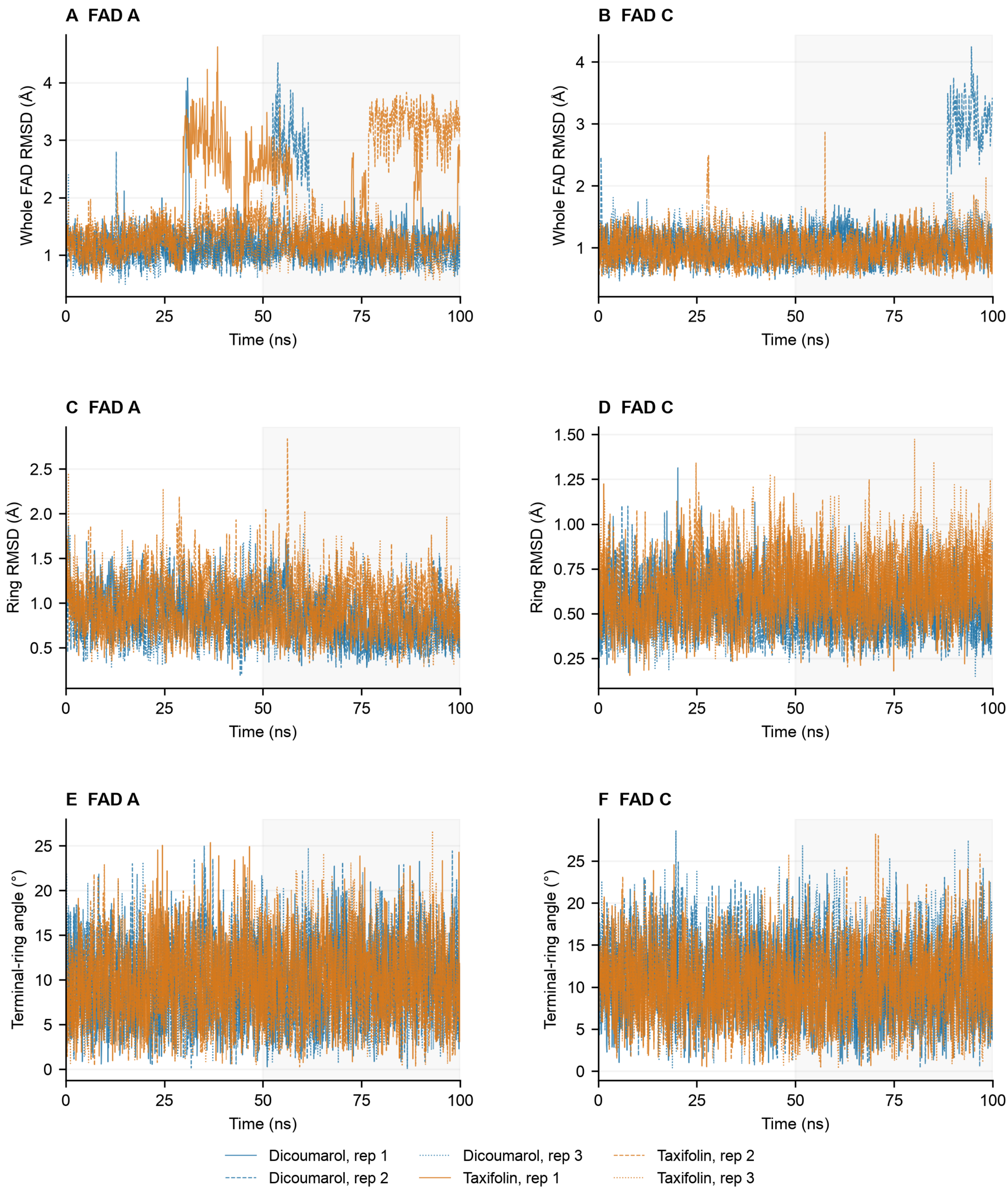

Fig H. NQO1 FAD behavior. The two FAD cofactors are shown separately by whole-cofactor and isoalloxazine-ring RMSD and terminal-ring angle. Late whole-cofactor changes should not be conflated with displacement of the ring. All independent replicas are displayed.

Fig I Within-target endpoint energy estimates

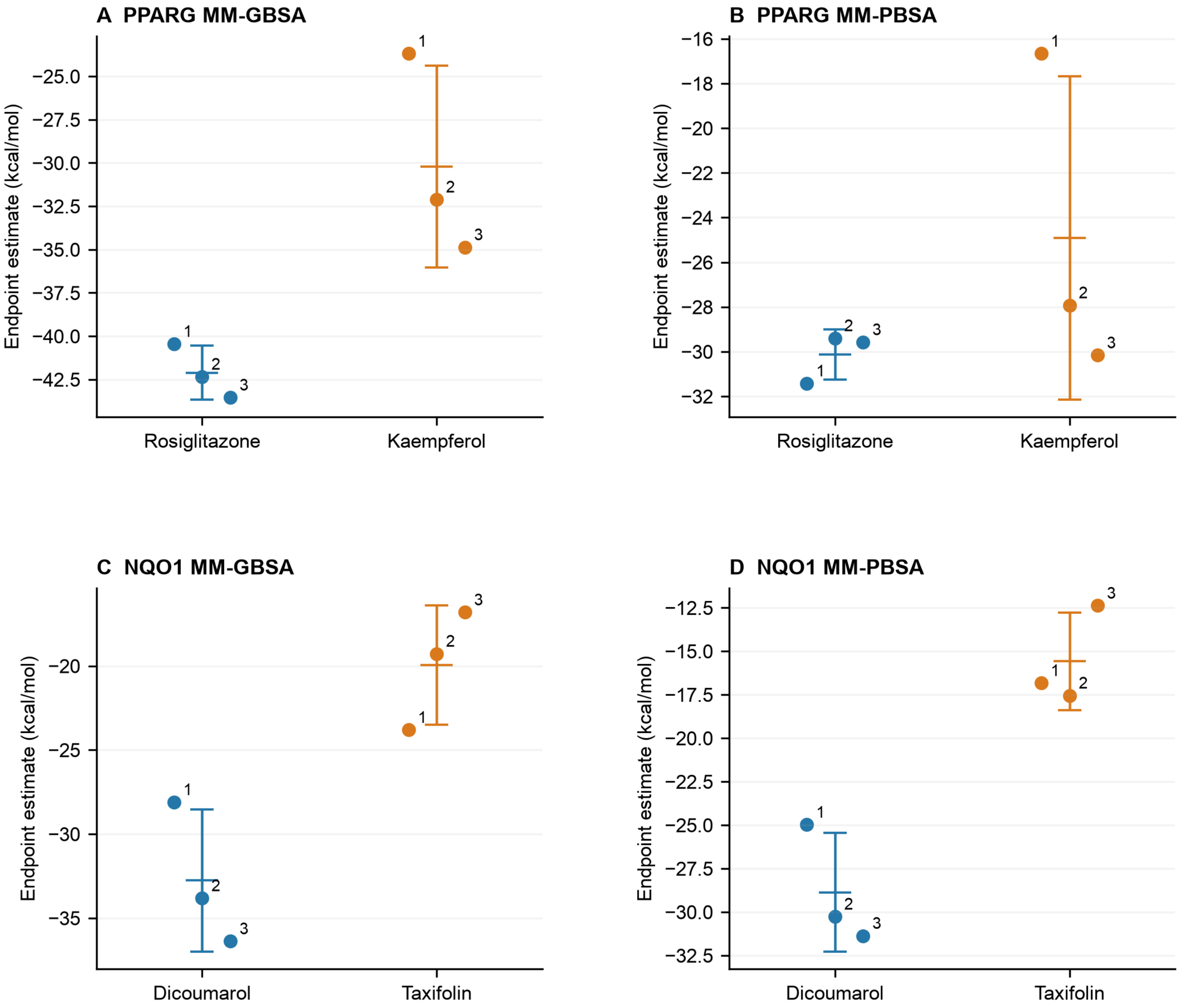

Dots: independent replicas; bars: mean ± between-replica SD. Entropy not included.

Fig I. Within-target endpoint energy estimates. Each point is an independent-replica mean from 101 fixed-window snapshots; bars indicate the mean of three replica means ± between-replica SD. PPARG and NQO1 are separate comparisons. MM-GBSA and MM-PBSA estimates omit conformational entropy and must not be interpreted as experimental binding free energies.
